# Inhibition of TGF-beta signaling protects from alpha-synuclein induced toxicity

**DOI:** 10.1038/s41420-025-02901-2

**Published:** 2025-12-12

**Authors:** Oscar Wing Ho Chua, Linghan Duan, Svenja Hanna Bothe, Valentin Evsyukov, Claudia Moebius, Marc Bickle, Günter U. Höglinger, Matthias Höllerhage

**Affiliations:** 1 Department of Neurology, Hannover Medical School, Hannover, Germany; 2 Center for Systems Neuroscience, Hannover, Germany; 3 HT-Technology Development Studio, Max Planck Institute of Molecular Cell Biology and Genetics, Dresden, Germany; 4 Department of Neurology, Ludwig-Maximilians-Universität (LMU) Munich, Munich, Germany; 5 German Center for Neurodegenerative Diseases (DZNE), Munich, Germany; 6 Munich Cluster for Systems Neurology (SyNergy), Munich, Germany

**Keywords:** Cell death in the nervous system, Parkinson's disease

## Abstract

Parkinson’s disease (PD) is histopathologically defined by the presence of Lewy bodies, which are intracellular proteinaceous inclusions that contain mainly aggregated alpha-synuclein (aSyn). It is believed that oligomeric intermediates between monomeric aSyn and large aggregates are neurotoxic, which would lead to the demise of dopaminergic neurons. Therefore, novel therapies preventing aSyn-induced cell death need to be developed. Therefore, we performed a genome-wide siRNA screening in an aSyn-induced dopaminergic cell death model and found the knockdown of three transforming growth factor-beta (TGFb) pathway-related genes to be protective. Hence, we hypothesized that a reduction in TGFb signaling would protect dopaminergic neurons from aSyn-induced toxicity. Thus, we validated the results of the genome-wide knockdown screening with the use of two different types of siRNAs. We confirmed that the knockdown of Activin receptor-like kinase 5 (*ALK5*) and Mothers against decapentaplegic homolog 2 (*SMAD2*), two genes of the TGFb pathway, protected dopaminergic neurons from aSyn-induced toxicity. An increase in TGFb signaling by treatment with TGFb ligands further exacerbated aSyn-induced toxicity, whereas this effect was mitigated by knockdown of *ALK5*, *SMAD2*, or Dynein light chain roadblock type-1 (*DYNLRB1*). Moreover, TGFb ligand treatment induced an up-regulation of *SNCA* mRNA expression in aSyn-overexpressing cells. Interestingly, consistent with the literature, we identified an up-regulation of the genes of the TGFb pathway in aSyn-overexpressing cells. Altogether, we identified a potential protective role by interference with the TGFb pathway against aSyn-induced toxicity. These findings provide a rationale for the development of novel strategies against PD.

## Introduction

Parkinson’s disease (PD) is the second-most prevalent neurodegenerative disorder. In PD, the death of dopaminergic neurons in the substantia nigra (SN) leads to a series of observable motor symptoms, such as bradykinesia, resting tremor, and rigidity [1]. Histopathologically, PD is defined by the presence of Lewy bodies (LBs) in the brains of PD patients [2]. LBs are intracellular proteinaceous inclusions that mainly contain abnormally aggregated alpha-synuclein (aSyn) [3, 4]. During the aggregation process of aSyn, oligomeric intermediates between monomers and large insoluble aggregates are formed, and these intermediates are believed to be toxic [5, 6]. We performed a genome-wide siRNA screening in dopaminergic Lund mesencephalic (LUHMES) cells [7, 8] with moderate overexpression of wild-type (WT) aSyn [9], in which the knockdown of 12 genes was identified to protect from degeneration [10]. Among these, three genes are related to the transforming growth factor-beta (TGFb) canonical pathway, namely Activin A receptor type 1C (*ACVR1C*, also known as Activin receptor-like kinase 7, *ALK7*), Dynein light chain roadblock type-1 (*DYNLRB1*), and Glycogen synthase kinase 3 beta (*GSK3B*).

**Fig. 1:**
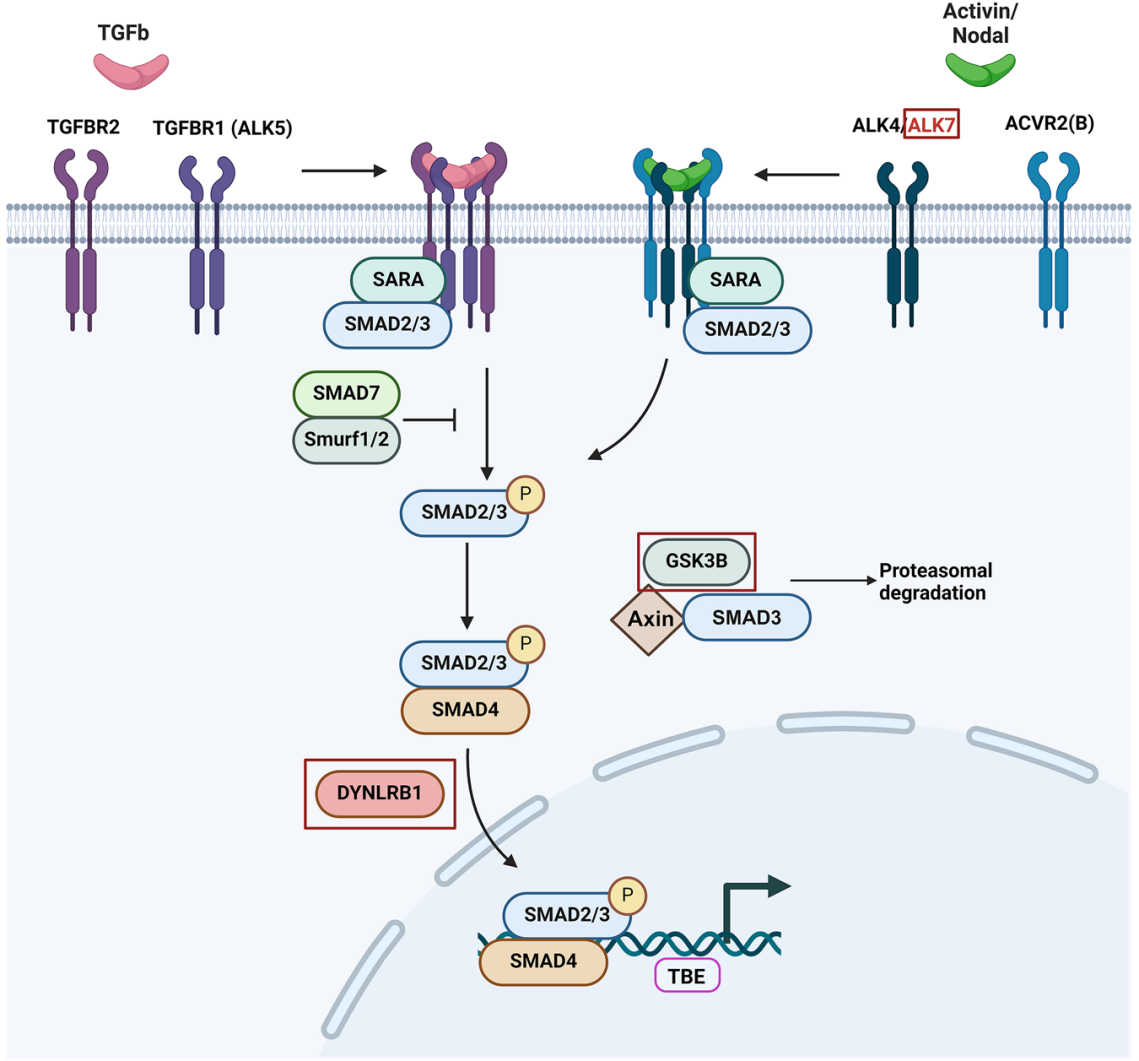
Schematic overview of the TGF pathway. Upon ligand binding (TGFb/Activin/Nodal), respective type-II receptor dimers of the TGFb pathway are activated, recruiting and activating respective type-I receptor dimers. Together, they form a heterotetrameric receptor complex that is internalized into the cell. SMAD2 and SMAD3 proteins are presented to the receptor complex via SMAD anchor for receptor activation (SARA). Subsequently, the SMAD2/3 proteins are phosphorylated and are translocated into the nucleus together with SMAD4, where the SMAD complex can bind to various target genes and drive target gene transcription via the transcription factor-binding element (TBE). The TGFb pathway can be regulated by, for example, competitive binding of the inhibitory SMAD7 protein to the receptor complex, or by proteasomal degradation of receptors or SMAD proteins via Smurf1/2 E3 ubiquitin ligases. ALK7 is one of the seven type-I receptors of the TGFb pathway that is specific for the ligand Nodal. DYNLRB1 is a member of the dynein light-chain family that was reported to be closely associated with SMAD2 nuclear translocation. On the other hand, GSK3B has been reported to be involved in the degradation of SMAD3. Created in BioRender. Chua, W. (2022) BioRender.com/e97d354.

The TGFb superfamily is one of the largest signaling pathway families in humans, comprising 33 ligands, 5 type-II receptors, and 7 type-I receptors. Pathways of the TGFb superfamily can be classified according to the ligands, as well as by the downstream cascades. The pathway that involves the phosphorylation of SMAD proteins is known as the canonical pathway [11] (Fig. 1). The binding of TGFb ligands (3 isoforms in humans) to the dimeric TGFb type-II receptor (TGFBR2) activates the TGFb canonical pathway. Upon ligand binding, TGFBR2 recruits and phosphorylates the dimeric TGFb type-I receptor (TGFBR1, also known as activin receptor-like kinase 5, ALK5) [11–13]. Together they form a heterotetrameric complex that is internalized into early endosomes, where the heterotetrameric receptor complex can phosphorylate Mothers against decapentaplegic homolog 2 (SMAD2) and/or SMAD3 proteins [12, 14, 15]. The activated SMAD2/3 complex is then coupled to SMAD4 for nuclear translocation, where the SMAD complex binds its target genes and drives their expression together with a number of different transcription factors [16]. ALK7 and DYNLRB1 are closely related to the TGFb pathway, as ALK7 is a type-I receptor that specifically transduces signals from the ligand Nodal [17], and DYNLRB1 is involved in the nuclear translocation of phospho-SMAD2 [18]. GSK3B has been implicated to be involved in the degradation of SMAD3 [19]. An illustration depicting the TGFb pathway can be found in Fig. 1.

In the central nervous system, TGFb ligands and receptors are expressed in most parts of the brain, and they are reported to be involved in multiple cellular processes. It has been shown that TGFb ligand treatment could promote the survival and differentiation of primary mesencephalic dopaminergic neurons [20, 21]. Moreover, TGFb signaling has been implicated to play a role in the regulation of neuronal proliferation and apoptosis [22, 23]. Due to its large distribution and involvement in multiple cellular processes, several studies looked into whether an increase in TGFb signaling could protect dopaminergic neurons from the toxicity induced by the mitochondrial toxin 1-methyl-4-phenylpyridinium (MPP^+^), both in vivo and in vitro [24–26]. However, results from these studies remain contradictory, and the role of TGFb signaling in the context of MPP^+^-induced toxicity remains inconclusive. Interestingly, one study has highlighted the therapeutic potential of TGFb signaling to rescue midbrain dopaminergic neurons against apoptosis [27].

In a previous work, our group discovered that the knockdown of genes involved in the TGFb signaling pathway protected LUHMES cells against aSyn-induced toxicity [10]. Hence, in this project, we aimed to investigate the role of the TGFb pathway upon aSyn overexpression.

## Results

### Validation of the results of the genome-wide siRNA screening

We validated whether the knockdown of *ALK7*, *DYNLRB1*, and *GSK3B* could protect cells from aSyn-induced toxicity in our standard cell culture conditions. Utilizing esiRNAs, as employed in the genome-wide screening, we aimed to validate the screening results. In addition, we used siPOOLs siRNAs to further confirm and consolidate our findings. Both types of siRNAs are ‘pools’ of siRNAs that are produced by enzymatic cleavage of long double-stranded RNA, in which these siRNAs all target the same mRNA. The use of these two distinct siRNA pools helped enhance the reliability of our outcomes.

**Fig. 2:**
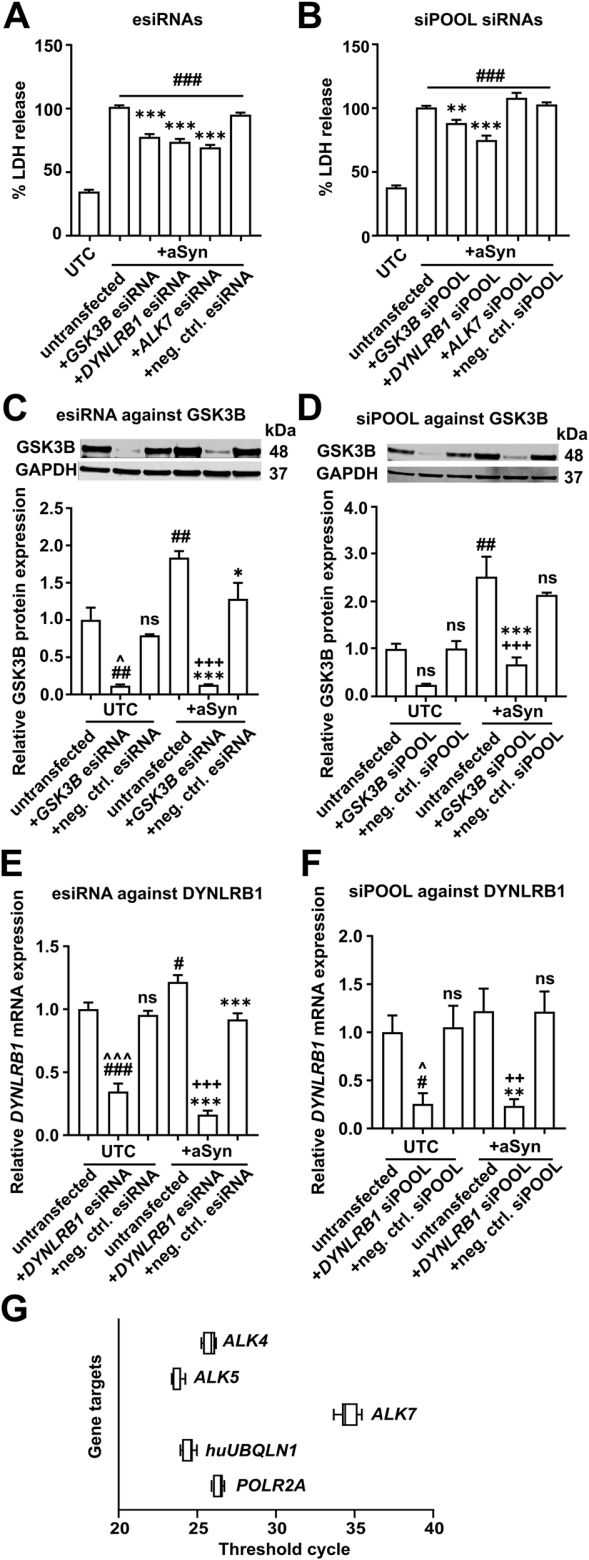
Validation of the genome-wide siRNA screening. Measurement of LDH release into the cell culture medium confirmed the protection against aSyn-induced cytotoxicity by esiRNAs (**A**) or siPOOL siRNAs (**B**) against *GSK3B*, *DYNLRB1*, and *ALK7*. Data are normalized to the LDH release of untransfected aSyn-overexpressing cells. Representative Western blots with an antibody against GSK3B confirmed the knockdown of GSK3B after transfection with the esiRNA (**C**) and the siPOOL siRNA (**D**) against *GSK3B* in untransduced cells (UTCs) and in aSyn-overexpressing cells. Full-size blot images of **C** and **D** can be found in Supplementary document 2, **A** and **B**, respectively. The knockdown efficacy of esiRNAs (**E**) and siPOOL siRNAs (**F**) against *DYNLRB1* was confirmed by qPCR analysis in UTCs and aSyn-overexpressing cells. **G** The plot shows the number of threshold cycles in the qPCR until which expression of the respective genes was detected in untransduced LUHMES cells. The expressions of *ALK4*, *ALK5*, and the two housekeeping genes were comparable (between 24th and 27th threshold cycle). The expression of *ALK7* was much lower in comparison to the other four genes in the chart, reaching the threshold value ~ 10 cycles later than the other four genes. UTCs: untransduced cells; aSyn: cells overexpressing alpha-Synuclein; *GSK3B* esiRNA: esiRNA against *GSK3B*; *DYNLRB1* esiRNA: esiRNA against *DYNLRB1*; *ALK7* esiRNA: esiRNA against *ALK7*; neg. ctrl. esiRNA: negative control esiRNA targeting firefly luciferase; *GSK3B* siPOOL: siPOOL siRNA against *GSK3B*; *DYNLRB1* siPOOL: siPOOL siRNA against *DYNLRB1*; *ALK7* siPOOL: siPOOL siRNA against *ALK7*; neg. ctrl. siPOOL: negative control siPOOL siRNA; untransfected: cells that were not transfected with siPOOL siRNAs. #*p* < 0.05, ##*p* < 0.01, ###*p* < 0.001 against untransfected UTCs; ^*p* < 0.05, ^^^*p* < 0.001 against UTCs + neg. ctrl. esiRNA/siPOOL; *p < 0.05, **p < 0.01, ***p < 0.001 against untransfected aSyn; +++*p* < 0.001 against aSyn + neg. ctrl. esiRNA/siPOOL; ns: not significant.

To analyze the changes in aSyn-related toxicity, LDH data were normalized relative to the LDH levels measured in aSyn-overexpressing cells. Thus, data from LDH released from aSyn-overexpressing cells are presented as 100%. We first validated the findings of the genome-wide siRNA screening [10] utilizing esiRNAs targeting *ALK7*, *DYNLRB1*, and *GSK3B*, which were highlighted in the primary screening. Notably, treatment with esiRNAs against *ALK7*, *DYNLRB1*, and *GSK3B* significantly protected LUHMES cells from aSyn-induced cytotoxicity (Fig. 2A). This reduction was evident in the diminished LDH release as compared to untreated aSyn-overexpressing cells (esiRNA against *ALK7*: 68.3 ± 2.2%, *p* < 0.001; esiRNA against *DYNLRB1*: 72.6 ± 2.6%, *p* < 0.001; esiRNA against *GSK3B*: 76.5 ± 2.5%, *p* < 0.001; each compared to untransfected aSyn-overexpressing cells: 100 ± 1.5%). By using the siPOOL siRNAs, we also confirmed that the transfection with siPOOL siRNAs against *DYNLRB1* and *GSK3B* protected aSyn-overexpressing cells (Fig. 2B), as quantified by the reduction in LDH released from aSyn-overexpressing cells (siPOOL siRNA against *DYNLRB1*: 74.3 ± 3.8%, *p* < 0.001; siPOOL siRNA against *GSK3B*: 87.7 ± 2.9%, *p* < 0.01; each compared to untransfected aSyn-overexpressing cells; 100 ± 1.6%). However, in contrast to the esiRNA, the treatment with the siPOOL siRNA against *ALK7* did not protect aSyn-overexpressing cells (Fig. 2B).

To determine whether the protection against aSyn-induced toxicity was the consequence of a specific knockdown of the respective target genes or an off-target effect, we investigated the expression of GSK3B, DYNLRB1, and ALK7 in untransduced cells and aSyn-overexpressing cells after the transfection with the siRNAs. By performing WB analyses, we confirmed that the esiRNA against *GSK3B* led to a significant down-regulation of GSK3B protein in both untransduced cells and aSyn-overexpressing cells (Fig. 2C; full-size Western blot in Supplementary File 2 Fig. A). Similarly, the siPOOL siRNA against *GSK3B* markedly reduced the expression of GSK3B protein in both untransduced cells and aSyn-overexpressing cells (Fig. 2D; full-size Western blot in Supplementary File 2 Fig. B). To reinforce our hypothesis that reducing GSK3B levels protects LUHMES cells from aSyn-induced toxicity, we tested treated LUHMES cells with two different inhibitors of GSK3B, tideglusib [28] and GSK3B inhibitor-VIII (GSK8) [29]. Both tideglusib and GSK8 led to a significant reduction in LDH release in aSyn-overexpressing cells, demonstrating their protective efficacy. Tideglusib reduced aSyn-induced toxicity at concentrations of 10 µM (88.8 ± 1.8, *p* < 0.01) and 20 µM (72.9 ± 3.3%, *p* < 0.001; untreated aSyn-overexpressing cells: 100 ± 2.03%; Supplementary Fig. S1A). Similarly, GSK8 treatment resulted in a significant reduction of LDH release in aSyn-overexpressing cells (100 ± 2.9%) at concentrations of 1 µM (84.4 ± 4.3%, *p* < 0.01) and 2 µM (57.4 ± 2.8%, *p* < 0.001; Supplementary Fig. S1B). Additionally, aSyn overexpression significantly elevated GSK3B levels as compared to untransduced cells, highlighting the role of GSK3B in aSyn-induced toxicity in our cell model. Collectively, our results suggest that reducing GSK3B availability, either through the knockdown of GSK3B or by direct pharmacological inhibition, protects LUHMES cells from aSyn-induced toxicity.

Due to the lack of commercially available WB antibodies for DYNLRB1, we assessed the knockdown efficacy of both the esiRNAs and the siPOOL siRNAs against *DYNLRB1* by qPCR. The esiRNA against *DYNLRB1* significantly reduced *DYNLRB1* mRNA expression in both untransduced cells and aSyn-overexpressing cells (Fig. 2E). We also identified a significant up-regulation of *DYNLRB1* associated with aSyn overexpression. Similarly, the siPOOL siRNA targeting *DYNLRB1* led to a significant reduction in *DYNLRB1* mRNA expression in untransduced cells and aSyn-overexpressing cells (Fig. 2F), indicating that both siRNAs against *DYNLRB1* were effective.

Since transfection with the siPOOL siRNA against *ALK7* did not protect against aSyn-induced toxicity, we conducted a deeper analysis of *ALK7* expression in LUHMES cells. In contrast to the gene expression of the other two type-I receptors of the TGFb pathway (*ALK4* and *ALK5*), as well as the housekeeping genes (*huUBQLN1* and *POLR2A*), *ALK7* mRNA was only detectable after 34.6 ± 0.2 cycles, indicating extremely low expression levels in LUHMES cells (Fig. 2G). Subsequently, we examined whether the esiRNA and the siPOOL siRNA targeting *ALK7* could reduce *ALK7* expression.

**Fig. 3:**
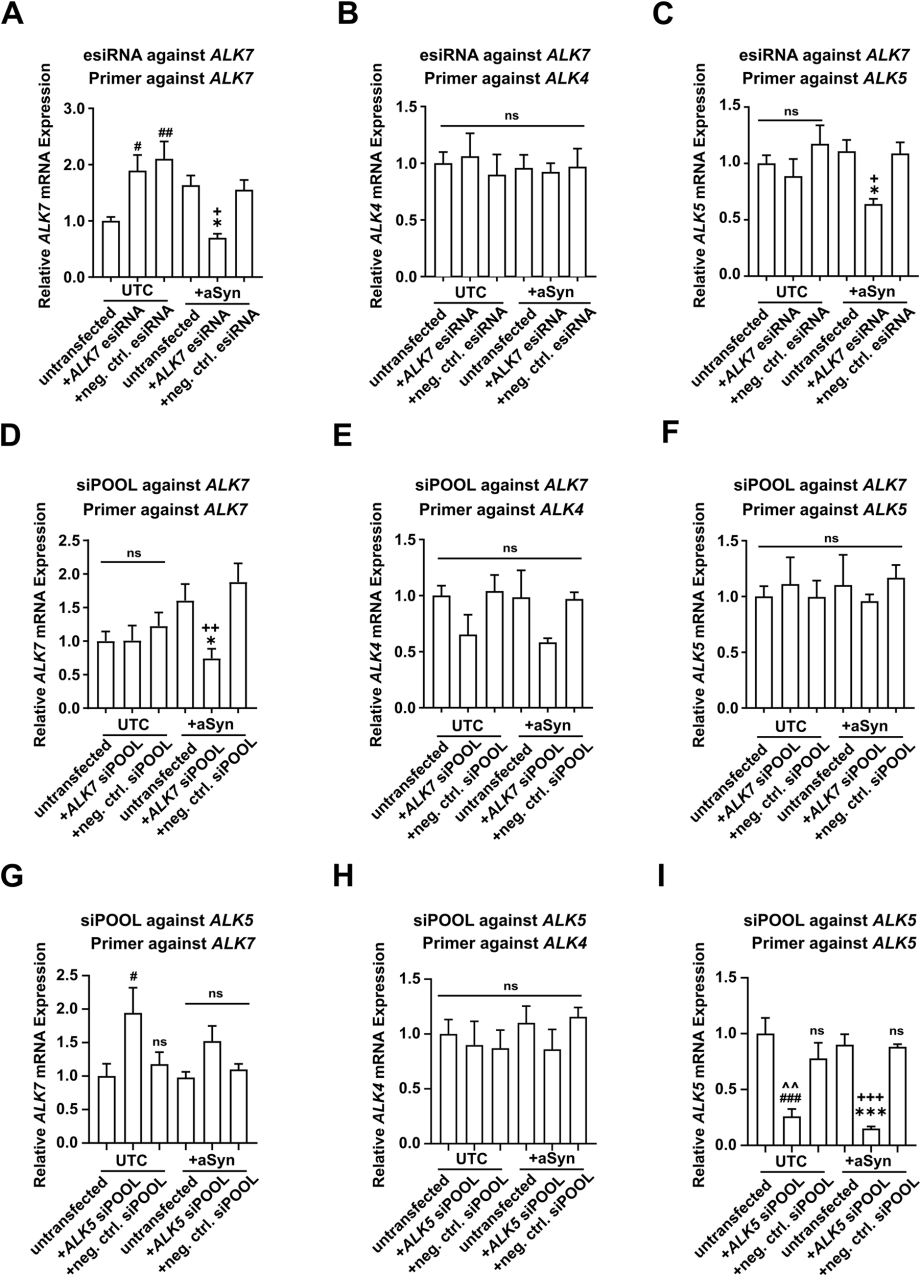
Confirmation of the efficacy of ALK7 knockdown. Quantification of the relative mRNA expression of *ALK7* (**A**), *ALK4* (**B**), and *ALK5* (**C**) after transfection with the esiRNA against *ALK7*. The esiRNA against *ALK7* led to a knockdown of *ALK7* (**A**) and *ALK5* (**C**) only in aSyn-overexpressing cells, but not in untransduced cells (UTCs). Quantification of the relative mRNA expression of *ALK7* (**D**), *ALK4* (**E**), and *ALK5* (**F**) after transfection with the siPOOL against *ALK7*. The siPOOL against *ALK7* led to a knockdown of *ALK7* only in aSyn-overexpressing cells but not in UTCs. Quantification of the relative mRNA expression of *ALK7* (**G**), *ALK4* (**H**), and *ALK5* (**I**) after transfection with the siPOOL against *ALK5*. The siPOOL against *ALK5* only led to a knockdown of ALK5 in both UTCs and aSyn-overexpressing cells. UTCs: untransduced cells; aSyn: cells overexpressing alpha-Synuclein; *ALK7* esi: esiRNA against *ALK7*; neg. ctrl. esiRNA: negative control esiRNA targeting firefly luciferase; *ALK7* siPOOL: siPOOL siRNA against *ALK7*; neg. ctrl. siPOOL: negative control siPOOL siRNA; *ALK5* si: siPOOL siRNA against *ALK5*; untransfected: cells that were not transfected with siPOOL siRNAs. #*p* < 0.05, ##*p* < 0.01, ###*p* < 0.001 against untransfected UTCs; ^^*p* < 0.01 against UTCs + neg. ctrl. esiRNA/siPOOL; *p < 0.05, ***p < 0.001 against untransfected aSyn; +*p* < 0.05, ++*p* < 0.01, +++*p* < 0.001 against aSyn + neg. ctrl. esiRNA/siPOOL; ns: not significant.

The esiRNA against *ALK7* significantly reduced *ALK7* expression only in aSyn-overexpressing cells (Fig. 3A). As the treatment with the esiRNA, but not the siPOOL siRNA against *ALK7*, conferred protection to LUHMES cells against aSyn-induced toxicity, we hypothesized that the protectiveness observed with the esiRNA against *ALK7* might arise from off-target activities. Considering the close phylogenetic similarity between ALK7 and the TGFb pathway receptors ALK4 and ALK5 [30–32], we hypothesized that *ALK4* and *ALK5* might be potential off-targets of the *ALK7* esiRNA. However, our analysis revealed that the esiRNA against *ALK7* did not down-regulate *ALK4* mRNA levels in either untransduced cells or aSyn-overexpressing cells, suggesting that *ALK4* was not the off-target of the *ALK7* esiRNA (Fig. 3B). In contrast, the esiRNA against *ALK7* significantly reduced *ALK5* mRNA expression in aSyn-overexpressing cells (Fig. 3C), indicating that *ALK5* was indeed an off-target.

The siPOOL siRNA against *ALK7* also resulted in a reduction in *ALK7* expression only in aSyn-overexpressing cells (Fig. 3D). However, unlike the esiRNA, the siPOOL siRNA against *ALK7* did not lead to significant changes in the expression of both *ALK4* and *ALK5* in either untransduced cells or aSyn-overexpressing cells (Fig. 3E, F). Given that the protection against aSyn-induced toxicity was only observed after the transfection of esiRNA against *ALK7*, for which we identified *ALK5* as off-target, together with the fact that the expression of *ALK7* was very low in LUHMES cells (Fig. 2G), we proposed that *ALK5* knockdown, rather than *ALK7* knockdown, was responsible for the observed protective effect against aSyn-induced toxicity.

### ALK5 knockdown and its downstream pathway protected LUHMES cells from aSyn-induced toxicity

**Fig. 4:**
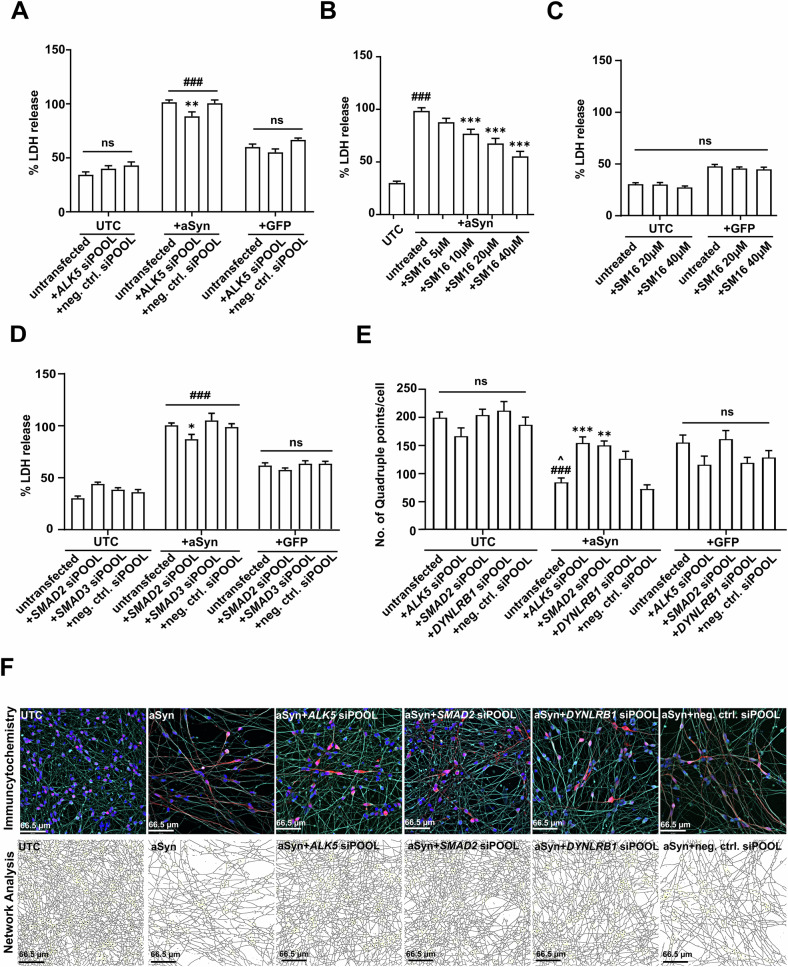
Knockdown of TGFb pathway genes protected LUHMES cells from aSyn-induced toxicity. **A** Quantification of LDH released into the cell culture medium as a measure for cytotoxicity showed that knockdown of *ALK5* by the siPOOL siRNA protected from aSyn-induced toxicity. In untransduced cells (UTCs) or GFP-expressing cells, the knockdown of *ALK5* did not change toxicity. Data are normalized to the LDH release of untransfected aSyn-overexpressing cells. Quantification of LDH release into the cell culture medium after treatment of LUHMES cells with SM16, an ALK5 inhibitor. SM16 treatment protected against aSyn-induced toxicity at concentrations of 10, 20, and 40 µM (**B**). In UTCs or GFP-expressing control cells, 20 and 40 µM of SM16 had no effect on cell viability (**C**). Data are normalized to the LDH release of untransfected aSyn-overexpressing cells. **D** Quantification of LDH release into the cell culture medium in UTCs, aSyn-overexpressing, and GFP-expressing cells after transfection with siRNAs against SMAD2 and SMAD3. The knockdown of *SMAD2* by siPOOL siRNA protected against aSyn-induced toxicity, whereas the knockdown of *SMAD3* did not protect against aSyn-induced toxicity. Data are normalized to the LDH release of untransfected aSyn-overexpressing cells. **E** Quantification of the number of quadruple points per cell as a measure for changes in the density of neuronal network showed that aSyn-overexpression led to a reduction in the density of neuronal network. Such reduction could be mitigated by the knockdown of *ALK5*, *SMAD2*, and *DYNLRB1*. On the other hand, the knockdown of *ALK5*, *SMAD2*, and *DYNLRB1* in UTCs or GFP-expressing cells did not result in changes in the neuronal network complexity. **F** Representative images of the ICC staining against DAPI (blue), GFP (green, if any), aSyn (red), and beta-III tubulin (cyan) were obtained. Corresponding network analysis for each representative figure is shown below the staining, in which black lines outline the neurites. UTCs: untransduced cells; aSyn/+aSyn: cells overexpressing alpha-Synuclein; +GFP: cells expressing GFP; *ALK5* siPOOL: siPOOL siRNA against *ALK5*; neg. ctrl. siPOOL: negative siPOOL siRNA; *SMAD2* siPOOL: siPOOL siRNA against *SMAD2*; *SMAD3* siPOOL: siPOOL siRNA against *SMAD3*; untreated: cells that were not treated with SM16; untransfected: cells that were not transfected with siPOOL siRNAs. ###*p* < 0.001 against untransfected UTCs; *p < 0.05, **p < 0.01, ***p < 0.001 against untransfected aSyn-overexpressing cells. ns: not significant across the compared groups.

To verify that the knockdown of *ALK5* protected against aSyn-induced toxicity, we first validated whether the siPOOL siRNA against *ALK5* could effectively and specifically reduce *ALK5* mRNA expression. The siPOOL siRNA against *ALK5* significantly reduced *ALK5* expression in both untransduced cells and aSyn-overexpressing cells (Fig. 3I). We also confirmed that the siPOOL siRNA against *ALK5* did not lead to changes in the mRNA expression of *ALK4* or *ALK7* in both untransduced cells and aSyn-overexpressing cells (Fig. 3G, H). We then confirmed that the knockdown of *ALK5* could protect LUHMES cells from aSyn-induced toxicity (Fig. 4A). The knockdown of *ALK5* protected LUHMES cells from aSyn-induced toxicity, as quantified by a reduction of LDH release (from 100 ± 2.0% as in untreated aSyn-overexpressing cells to 87.2 ± 4.0%, *p* < 0.01). The protective effect of *ALK5* knockdown was specific against aSyn-induced toxicity, since the knockdown of *ALK5* did not lead to changes in cell viability in either untransduced cells or GFP-expressing cells (Fig. 4A).

Next, we used an inhibitor of ALK5, SM16 [33, 34], to further confirm if the inhibition of ALK5 protected against aSyn-induced toxicity (Fig. 4B). As anticipated, SM16 treatment led to a significant dose-dependent protection against aSyn-induced toxicity. In particular, SM16 treatment led to a reduction of LDH release in aSyn-overexpressing cells from 100 ± 2.2% to 77.5 ± 4.2% at 10 µM (*p* < 0.001), to 68.1 ± 4.8% at 20 µM (*p* < 0.001), and to 55.6 ± 4.8% at 40 µM (*p* < 0.001). Similar to the observation made after the knockdown of *ALK5*, the protection by the treatment with SM16 was specific to aSyn-overexpressing cells (Fig. 4C).

We then explored whether the knockdown of *SMAD2* and/or *SMAD3*, the downstream targets of ALK5, could confer protection against aSyn-induced toxicity. Interestingly, only the knockdown of *SMAD2*, but not *SMAD3*, demonstrated a protective effect. When compared to the LDH released from untreated aSyn-overexpressing cells, the knockdown of *SMAD2* led to a reduction of LDH release from 100 ± 3.4% to 88.2 ± 4.6% (*p* < 0.05). Furthermore, the protection after the knockdown of *SMAD2* was only observed in aSyn-overexpressing cells, indicating an aSyn-dependent effect (Fig. 4D). Due to the high sequence homology between *SMAD2* and *SMAD3* [35, 36], we verified the specificities of siPOOL siRNAs for their respective targets. In both untransduced cells and aSyn-overexpressing cells, we found that the siPOOL siRNA against *SMAD2* specifically reduced *SMAD2* expression without affecting the expression of *SMAD3* (Supplementary Fig. S1C). In the same manner, the siPOOL siRNA against *SMAD3* specifically reduced *SMAD3* expression without affecting the expression of *SMAD2* (Supplementary Fig. S1D). Interestingly, the expression of *SMAD2* was much higher than *SMAD3* (~10 threshold cycles higher, i.e., over two orders of magnitude) in LUHMES cells (Supplementary Fig. S1E).

Since the TGFb pathway is involved in the differentiation of dopaminergic neurons [24, 37], we performed a control experiment to verify that the knockdown of *ALK5*, *SMAD2*, *SMAD3*, or *DYNLRB1* did not interfere with the differentiation of LUHMES cells. We confirmed that the mRNA expression of tyrosine hydroxylase (*TH*), a dopaminergic neuronal marker, was not significantly altered by the knockdown of *ALK5*, *SMAD2*, *SMAD3*, or *DYNLRB1* in untransduced cells, aSyn-overexpressing cells, and GFP-expressing cells (Supplementary Fig. S1F). To further ensure that the knockdown of *ALK5*, *SMAD2*, *SMAD3*, or *DYNLRB1* did not impact the transcriptional activities of the adenoviral vectors used in our experiments, we measured the expression of aSyn on both transcript and protein levels. The knockdown of *ALK5*, *SMAD2*, *SMAD3*, or *DYNLRB1* did not affect the expression of *SNCA*, the gene that encodes aSyn, nor the level of aSyn protein (Supplementary Fig. S1G, H; full-size Western blot in Supplementary File 2 Fig. C) in untransduced cells, aSyn-overexpressing cells, or GFP-expressing cells. Concurrently, the knockdown of *ALK5*, *SMAD2*, *SMAD3*, or *DYNLRB1* did not interfere with the expression of GFP in GFP-expressing cells (Supplementary Fig. S1I). These data emphasize that the protection against aSyn-induced toxicity resulting from the knockdown of *ALK5*, *SMAD2*, or *DYNLRB1* was not caused by an interference with differentiation of LUHMES cells, or by a change in the transcriptional activities of the adenoviral vectors. In addition, as *SMAD3* expression was low in LUHMES cells and its knockdown did not yield observable phenotypic changes such as protection against aSyn-induced toxicity (Fig. 4D) or changes in the expression of aSyn (Supplementary Fig. S1G, H), we excluded *SMAD3* in the following experiments.

We then performed immunocytochemistry to investigate whether the knockdown of *ALK5*, *SMAD2*, and *DYNLRB1* also ameliorated loss of neuronal network density (Fig. 4F; untransduced cells: Supplementary Fig. S2A; GFP-expressing cells: Supplementary Fig. S2B). We quantified the density of the neuronal network by counting the number of quadruple points per cell (Fig. 4E). Each quadruple point reflects a dendritic junction that contains or connects four distinct neurite branches, serving as an indicator of neuronal network complexity. In untransduced cells and GFP-expressing cells, the knockdown of *ALK5*, *SMAD2*, or *DYNLRB1* did not significantly alter neuronal network density, as compared to respective control cells without siRNA transfection (Fig. 4G). As expected, aSyn overexpression substantially decreased the density of the neuronal network, as quantified by a reduction in the number of quadruple points per cell in comparison to the untransduced cells. (untransduced cells: 199.7 ± 10.7; aSyn-overexpressing cells: 84.6 ± 8.5; *p* < 0.001). Such reduction of the neuronal network complexity in aSyn-overexpressing cells was counteracted by the knockdown of *ALK5* and *SMAD2*, as we observed a significant increase in the number of quadruple points per cell in comparison to the untransfected aSyn-overexpressing cells (*ALK5* knockdown: 154.7 ± 11.4, *p* < 0.001; *SMAD2* knockdown: 150.5 ± 8.4, *p* = 0.002; Fig. 4E). On the other hand, the knockdown of *DYNLRB1* also led to an increase in the density of neuronal network of aSyn-overexpressing cells, however it did not reach statistical significance (126.5 ± 13.2; Fig. 4F). To further support these findings, we also detected activated caspases 3/7, an apoptotic marker, using a fluorescent dye (CellEvent™; Supplementary Fig. S2C, D). In aSyn-overexpressing cells, the knockdown of *SMAD2* led to an obvious reduction in the expression of caspase 3/7 (from 100 ± 10.0% to 65.3 ± 5.73%).

In summary, we demonstrated that reducing TGFb signaling, specifically via the knockdown of *ALK5*, *SMAD2*, and *DYNLRB1*, protected LUHMES cells against aSyn-induced toxicity. In particular, the knockdown of ALK5 prevented the loss of neuronal network density, whereas the knockdown of *SMAD2* not only preserved neuronal network density but also inhibited apoptotic activation.

### The over-activation of TGFb pathway is detrimental to aSyn-overexpressing cells

**Fig. 5:**
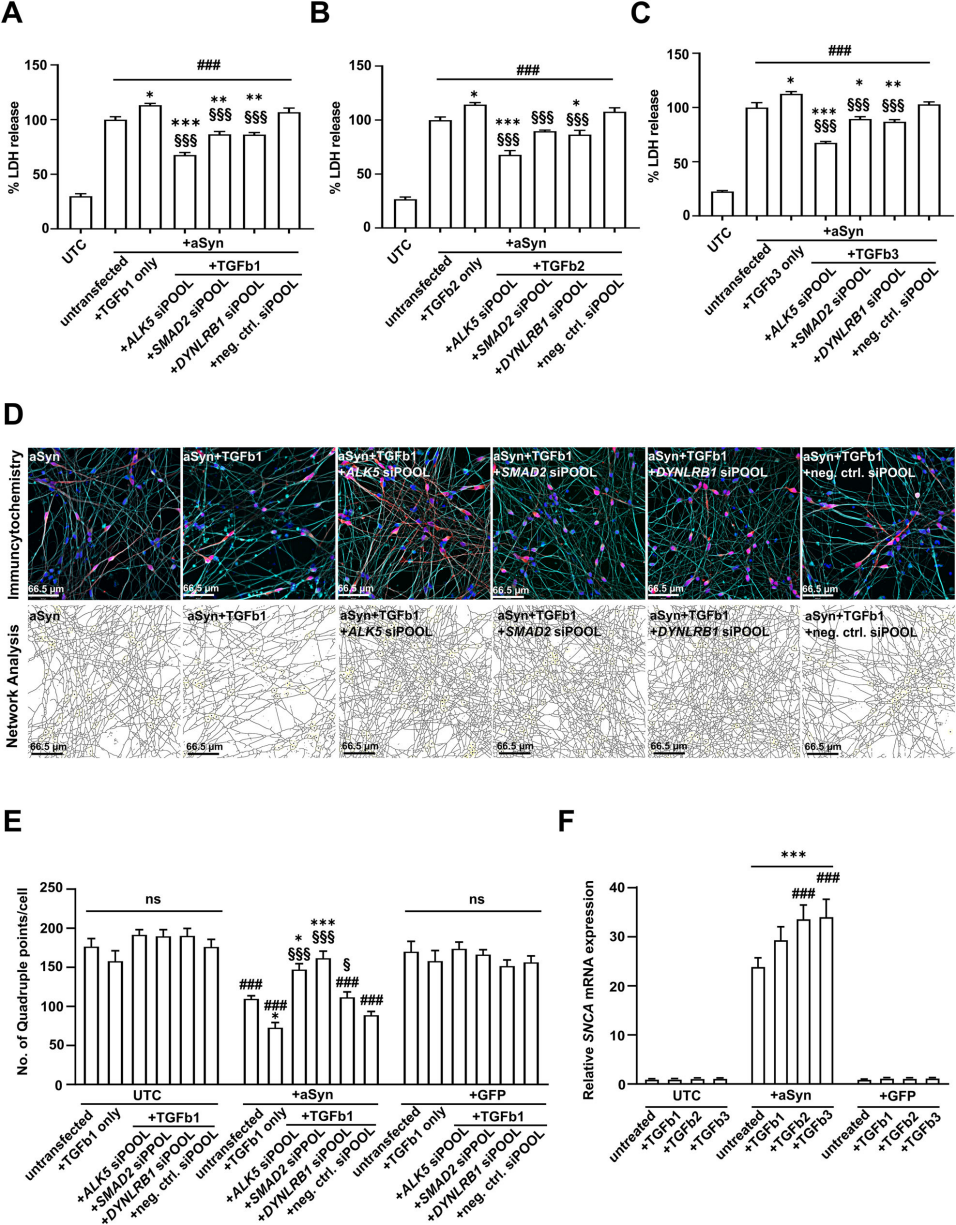
Stimulation of the TGFb pathway increased toxicity in aSyn-overexpressing cells. Quantification of LDH release into the cell culture medium after treatment of LUHMES cells with TGFb1 (**A**), TGFb2 (**B**), or TGFb3 (**C**). The treatment of TGFb ligands led to an increase in aSyn-induced toxicity, as compared to untreated aSyn-overexpressing cells. The knockdown of *ALK5*, *SMAD2*, and *DYNLRB1* by siPOOL siRNA significantly protected TGFb ligand-treated aSyn-overexpressing cells. Data are normalized to the LDH release of untreated aSyn-overexpressing cells. **D** Representative images of the ICC staining against DAPI (blue), GFP (green, if any), aSyn (red), and beta-III tubulin (cyan) in aSyn-overexpressing cells. Corresponding network analysis for each representative figure is shown below the staining, in which black lines outline the neurites. **E** Quantification of the number of quadruple points per cell as a measure for changes in the density of neuronal network showed that the treatment of TGFb1 led to a reduction in the density of neuronal network in aSyn-overexpressing cells. The knockdown of *ALK5*, *SMAD2*, or *DYNLRB1* by siPOOL siRNA significantly increased the density of the neuronal network in TGFb1-treated aSyn-overexpressing cells. TGFb1 ligand treatment did not result in significant changes in the density of neuronal network in untransduced cells (UTCs) or GFP-expressing cells. In addition, no significant changes in the density of the neuronal network resulted from the knockdown of *ALK5*, *SMAD2*, and *DYNLRB1* in TGFb1-treated UTCs or GFP-expressing cells. **F** Quantification of the relative mRNA expression of *SNCA* after the treatment of TGFb1, TGFb2, or TGFb3 in UTCs, aSyn-overexpressing cells, and GFP-expressing cells. TGFb2 and TGFb3 treatment led to a significant increase in the expression of *SNCA* only in aSyn-overexpressing cells. UTCs: untransduced cells; aSyn/+aSyn: cells overexpressing alpha-Synuclein; +GFP: cells expressing GFP; *ALK5* siPOOL: siPOOL siRNA against *ALK5*; neg. ctrl. siPOOL: negative control siPOOL siRNA; *SMAD2* siPOOL: siPOOL siRNA against *SMAD2*; *DYNLRB1* siPOOL: siPOOL siRNA against *DYNLRB1*; untreated: cells that were not treated with TGFb ligands; untransfected: cells that were not transfected with siPOOL siRNAs. #*p* < 0.05, ###*p* < 0.001 against untreated UTCs; *p < 0.05, **p < 0.01, ***p < 0.001 against untreated aSyn; §*p* < 0.05, §§§*p* < 0.001 against aSyn+TGFb ligand; ^*p* < 0.05 against untreated GFP; ns: not significant.

Since the inhibition of TGFb signaling protected against aSyn-induced toxicity, we explored whether activating this pathway would have the opposite effect. Therefore, we treated aSyn-overexpressing cells with recombinant human TGFb proteins. We used TGFb ligands at 10 ng/mL, a dose that was previously reported to effectively activate the TGFb pathway [38, 39]. In comparison to untreated aSyn-overexpressing cells (indicated as 100%), treatment with TGFb1, TGFb2, and TGFb3 led to an increase of LDH release in aSyn-overexpressing cells (TGFb1: 113.3 ± 2.0%, *p* < 0.05; TGFb2: 114.4 ± 2.2%, *p* < 0.05; TGFb3: 112.6 ± 2.5%, *p* = 0.01; Fig. 5A–C). Furthermore, the increase in cytotoxicity induced by TGFb ligand treatment was also reflected by a decrease in neuronal network density. Specifically, following the treatment with TGFb1, we observed a significant decrease in the number of quadruple points per cell, dropping from 109.9 ± 4.5 in untreated aSyn-overexpressing cells to 72.9 ± 7.2 (*p* = 0.03; Fig. 5D, E). Notably, this cytotoxic effect caused by TGFb ligand treatment was exclusive to aSyn-overexpressing cells. In contrast, untransduced or GFP-expressing cells treated with TGFb ligand (10 ng/mL) exhibited no significant changes in cell viability (Supplementary Fig. S3A, B) or neuronal network density (Fig. 5E, Supplementary Fig. S3D, E).

To gain further evidence for the role of TGFb signaling in aSyn-induced toxicity, we examined whether the increase in cytotoxicity after TGFb treatment could be mitigated by knocking down key genes in the TGFb signaling pathway. Therefore, aSyn-overexpressing LUHMES cells were treated with TGFb1, TGFb2, or TGFb3 and transfected with siPOOL siRNAs against *ALK5*, *SMAD2*, and *DYNLRB1*. As expected, the knockdown of *ALK5*, *SMAD2*, and *DYNLRB1* effectively ameliorated the increase in toxicity resulting from the treatment with TGFb ligands. In TGFb1-treated aSyn-overexpressing cells, a reduction in LDH release was observed upon the knockdown of *ALK5* (67.7 ± 2.6%, *p* < 0.001), *SMAD2* (86.6 ± 3.0%, *p* < 0.001), and *DYNLRB1* (86.4 ± 2.2%, *p* < 0.001), in comparison to the LDH released from TGFb1-treated aSyn-overexpressing cells (113.3 ± 2.0%; Fig. 5A). Similarly, in TGFb2-treated aSyn-overexpressing cells, a reduction in LDH release was observed upon the knockdown of *ALK5* (67.8 ± 4.3%, *p* < 0.001), *SMAD2* (89.8 ± 1.3%, *p* < 0.001), and *DYNLRB1* (86.6 ± 4.3%, *p* < 0.001), in comparison to the LDH released from TGFb2-treated aSyn-overexpressing cells (114.4 ± 2.2%; Fig. 5B). The same trend was also observed in TGFb3-treated aSyn-overexpressing cells, where a reduction in LDH release was observed upon the knockdown of *ALK5* (67.5 ± 1.6%, *p* < 0.001), *SMAD2* (89.5 ± 2.5%, *p* < 0.001) and *DYNLRB1* (86.9 ± 2.3%, *p* < 0.001), in comparison to TGFb3-treated aSyn-overexpressing cells (112.6 ± 2.5%; Fig. 5C). The decrease in cytotoxicity resulted from the knockdown of *ALK5*, *SMAD2*, and *DYNLRB1* was also reflected in the increase in the density of neuronal network of TGFb1-treated aSyn-overexpressing cells (Fig. 5D, E). Specifically, the knockdown of *ALK5* in TGFb1-treated aSyn-overexpressing cells led to a significant increase in the number of quadruple points per cell from 72.9 ± 7.2 to 147.4 ± 8.0 (*p* < 0.001), the knockdown of *SMAD2* to 161.9 ± 9.6 (*p* < 0.001), and the knockdown of *DYNLRB1* to 111.9 ± 7.5 (*p* = 0.02). In summary, with these experiments, we demonstrated that an increase in TGFb signaling via treatment with TGFb ligands exacerbates toxicity in aSyn-overexpressing cells, and this detrimental effect can be mitigated through the knockdown of *ALK5*, *SMAD2*, and *DYNLRB1*.

In addition, we also performed a staining of activated caspases 3/7 as an apoptotic marker to complement our findings (Supplementary Fig. S4A, B). Even though TGFb1 treatment in aSyn-overexpressing cells did not lead to a significant increase in the intensity of caspase 3/7 signals as compared to untreated aSyn-overexpressing cells (100 ± 7.3%), a significant decrease in caspase 3/7 signals was observed upon the knockdown of *ALK5* (65.0 ± 3.4%), *SMAD2* (57.8 ± 6.8%), or *DYNLRB1* (77.3 ± 1.3%), indicating a significant decrease in the number of apoptotic cells. These results further illustrate that the knockdown of TGFb signaling can effectively counteract the increase of aSyn-induced toxicity resulting from TGFb1 treatment.

To further understand why TGFb ligand treatment exacerbated aSyn-induced toxicity, we examined whether TGFb ligand treatment influenced *SNCA* expression. Interestingly, we observed that TGFb ligand treatment led to an additional up-regulation of the *SNCA* mRNA in aSyn-overexpressing cells (untreated aSyn-overexpressing cells: 24.0 ± 2.0; TGFb1 treatment: 29.5 ± 2.9, *p* = 0.07; TGFb2 treatment: 33.7 ± 3.0, *p* < 0.001; TGFb3 treatment: 34.1 ± 3.8, *p* < 0.001; Fig. 5F). In contrast, this treatment did not change the expression of the *SNCA* in untransduced cells or GFP-expressing cells. To determine if TGFb ligand treatment had an influence on the transcriptional activities of the adenoviral vectors, we then investigated if TGFb ligand treatment would also lead to an up-regulation of GFP in GFP-expressing cells. However, there was no significant increase in the expression of the GFP transcript following treatment with TGFb1, TGFb2, or TGFb3 in GFP-expressing cells (Supplementary Fig. S3C). These findings indicate that the up-regulation of *SNCA* induced by TGFb ligand treatment was specific to the aSyn-overexpressing condition and not due to an increase in the transcriptional activities of the adenoviral vectors.

### Transcripts of the TGFb pathway were up-regulated upon aSyn-overexpression

**Fig. 6:**
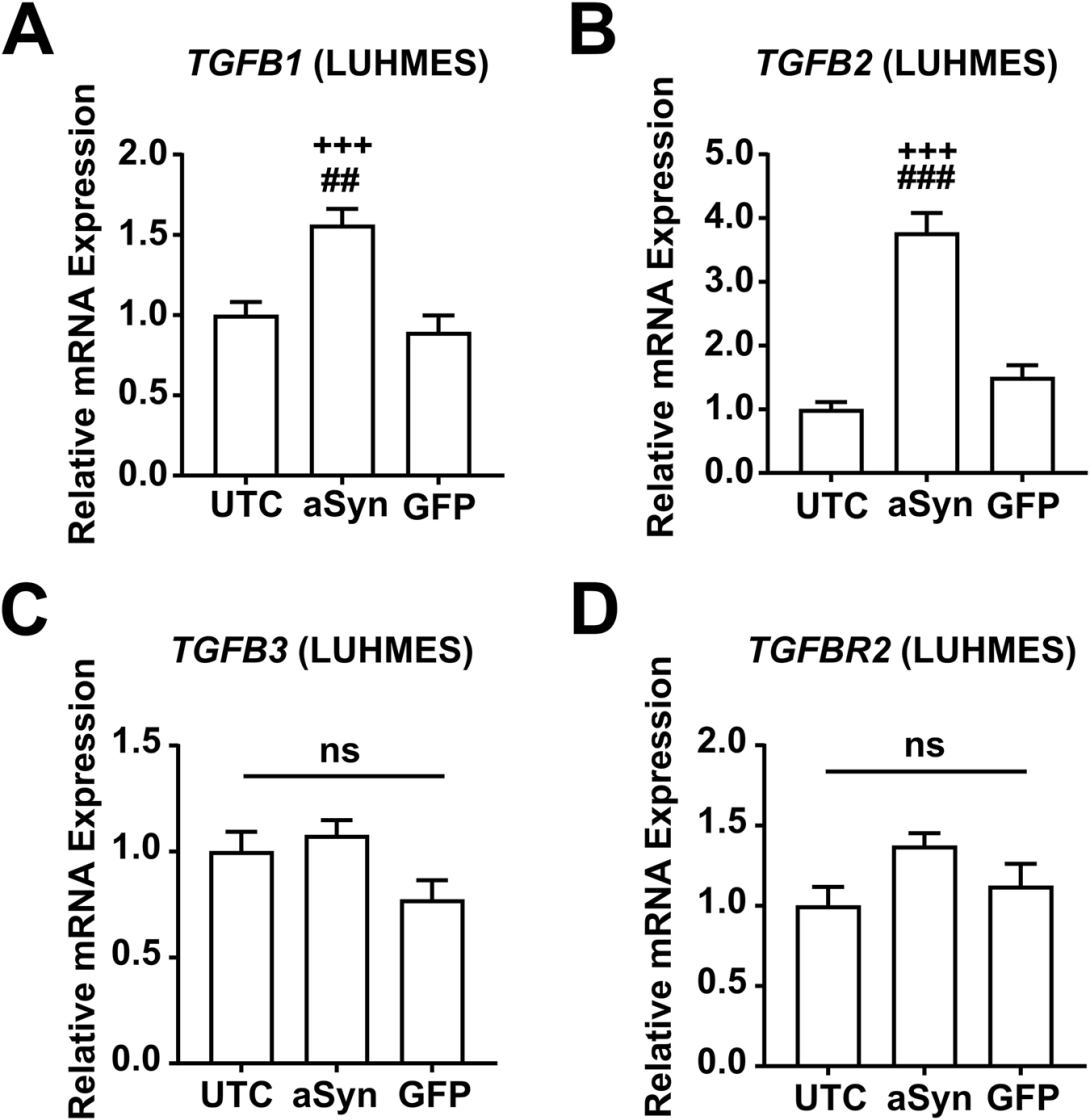
aSyn overexpression upregulated genes of the TGFb pathway in LUHMES cells. Quantification of the relative gene expression of *TGFB1* (**A**), *TGFB2* (**B**), *TGFB3* (**C**), and *TGFBR2* (**D**) was analyzed in untransduced cells (UTCs), aSyn-overexpressing cells, and GFP-expressing cells. *TGFB1* and *TGFB2* were found to be up-regulated in aSyn-overexpressing cells only. #*p* < 0.05, ##*p* < 0.01, ###*p* < 0.001 against UTCs; +++*p* < 0.001: *p* < 0.001 against GFP-expressing cells; ns: not significant.

Next, we examined whether the overexpression of aSyn led to changes in the expression of genes involved in the TGFb pathway. We have previously identified a significant increase in the expression of *SMAD2* (1.3 ± 0.1 fold, *p* = 0.05; Supplementary Fig. S1C) and *SMAD3* (4.6 ± 0.1 fold, *p* < 0.001, Supplementary Fig. S1D) in aSyn-overexpressing cells. In contrast, we did not observe an up-regulation of *ALK4* or *ALK5* in aSyn-overexpressing cells (Fig. 3). To expand upon these findings, we analyzed the expression levels of four additional key TGFb pathway genes, namely *TGFB1*, *TGFB2*, *TGFB3*, and *TGFBR2* in untransduced cells, aSyn-overexpressing cells, and GFP-expressing cells (Fig. 6). We found that aSyn-overexpressing cells, in comparison to untransduced cells, showed higher expression levels of *TGFB1* (1.6 ± 0.1 fold, *p* = 0.001) and *TGFB2* (3.8 ± 0.3 fold, *p* < 0.001). Interestingly, the expressions of *TGFB1*, *TGFB2*, *TGFB3*, and *TGFBR2* in untransduced LUHMES cells were comparable (between 28th and 32nd threshold cycle, Supplementary Fig. S3F). On the other hand, we did not observe any differences in the expression of genes of the TGFb pathway between untransduced cells and GFP-expressing cells (Fig. 6, Supplementary Fig. S3G). This suggests that TGFb signaling was up-regulated because of aSyn overexpression.

## Discussion

Previously, in a genome-wide siRNA screening of a PD cell model, we identified that the knockdown of three genes (*ALK7*, *DYNLRB1*, and *GSK3B*), which are associated with the TGFb signaling pathway, was found to mitigate aSyn-induced toxicity [10]. Hence, in this project, we investigated the role of the TGFb signaling pathway in aSyn-induced toxicity. Consistent with our initial findings in the siRNA screening, the knockdown of *DYNLRB1* and *GSK3B* by different knockdown paradigms (i.e., esiRNA and siPOOL siRNA) protected human dopaminergic neuron-like cells (LUHMES cells) from aSyn-induced toxicity. In addition, the treatment with two inhibitors of GSK3B, tideglusib and GSK8, also demonstrated a protective effect. On the other hand, while we identified that the transfection of the esiRNA provided protection against aSyn-induced toxicity, the siPOOL siRNA against *ALK7* did not. A deeper analysis revealed that the actual target of the *ALK7* esiRNA was *ALK5*, the gene coding for the type-I receptor of the TGFb pathway. We subsequently proved that the knockdown of *ALK5* safeguarded LUHMES cells from aSyn-induced toxicity. Furthermore, treatment with a specific ALK5 inhibitor (SM16) also conferred a similar protective effect. In addition to *ALK5*, we identified that the knockdown of *SMAD2*, a downstream target of ALK5, also protected against aSyn-induced toxicity.

In addition to this demonstration that the reduction of TGFb signaling was protective against aSyn-induced toxicity, we found that the opposite (i.e., stimulation of TGFb signaling by ligand treatment) was detrimental in our PD cell model. Furthermore, the knockdown of TGFb-associated genes abolished the adverse effects evoked by TGFb ligands. Moreover, we found that TGFb ligand treatment led to an increase in *SNCA* expression in our PD cell model, and aSyn-overexpression led to an up-regulation of *TGFB1*, *TGFB2*, *SMAD2*, and *SMAD3* in LUHMES cells.

In this project, we have provided compelling evidence that the reduction of TGFb signaling protected dopaminergic neurons from aSyn-induced toxicity, and an increase in TGFb signaling led to an elevation of aSyn-induced toxicity. Even though TGFb ligands were reported to be generally neurotrophic to dopaminergic neurons [24], few studies have explored the therapeutic potential of the TGFb signaling pathway in PD. In these toxin-based PD models, mouse or rat dopaminergic primary neurons or mice were treated with a mitochondrial complex I inhibitor MPP^+^ or 1-methyl-4-phenyl-1,2,3,6-tetrahydropyridine (MPTP; precursor of MPP^+^) that specifically kills dopaminergic neurons [40]. However, the outcomes varied. On one hand, one study reported that dopaminergic neurons in rat mesencephalic primary cultures that had been treated with TGFb1 were less susceptible to MPP^+^ [24]. In line with that, another study showed that an increase in TGFb signaling via the expression of a constitutively active ALK5 protected mouse dopaminergic neurons in the SN from MPTP-induced toxicity in vivo [26]. On the other hand, one study reported that the adenoviral overexpression of TGFb1 in the striatum led to a decrease in the number of midbrain dopaminergic neurons in MPTP-injected mice in vivo [25]. These conflicting outcomes highlight the uncertainty surrounding the role of TGFb signaling in toxin-based PD models. In distinction from previous studies, we investigated the role of TGFb signaling in a human PD cell model that shows toxicity induced by human aSyn. One advantage of our cell model is its close resemblance to neurons that degenerate in PD, providing a more accurate representation of the disease in humans. One study demonstrated that aSyn-toxicity models could replicate human PD pathology more closely than toxin models like 6-hydroxydopamine (6-OHDA) in vivo. The overexpression of aSyn resulted in a progressive loss of dopaminergic neurons that occurred in the course of eight weeks, accompanied by axonal swellings and an impairment in post-synaptic activity. These features were absent in 6-OHDA models [41]. Our human aSyn toxicity cell model enabled us to investigate how changes in the TGFb signaling led to changes in aSyn-induced cytotoxicity, as well as to observe how aSyn and the genes/proteins of the TGFb pathway could modulate the expression of each other. Importantly, we found that TGFb ligand treatment induced an up-regulation of the *SNCA* gene upon aSyn overexpression. Similar contradictions in neuroprotection were observed with glial cell-derived neurotrophic factor (GDNF), another member of the TGFb ligand family. While GDNF treatment was protective against MPTP-induced toxicity in mice and monkeys in vivo [42, 43], it failed to protect dopaminergic neurons against aSyn-induced toxicity in rats in vivo [44]. Furthermore, a recent clinical trial in which GDNF was infused into the putamen of PD patients via a skull-mounted transcutaneous port and four separate intraputamental infusion catheters also failed to meet its primary endpoint, as GDNF treatment did not lead to significant improvement of the motor symptoms in comparison to placebo control [45]. These results underscore the critical importance of selecting appropriate experimental models, as differences in experimental models could result in entirely opposite conclusions.

This study employed three distinct measurements to assess cell viability/cell death to verify whether the inhibition of TGFb signaling in aSyn-overexpressing cells could provide protection against aSyn-induced toxicity. Results from the LDH release assay and the network analysis indicated that suppressing TGFb signaling significantly reduced cell death in aSyn-overexpressing cells and simultaneously led to a significant increase in the density of the neuronal network. Furthermore, staining for activated caspases 3/7 confirmed the protective effect of reducing TGFb signaling. In TGFb1-treated aSyn-overexpressing cells, the knockdown of *ALK5*, *SMAD2*, and *DYNLRB1* all led to a significant reduction in the caspase signals. Even though all three assays assess cell death or cell viability, they differ fundamentally. LDH assay measures LDH released into the culture medium, which reflects membrane integrity; the network analysis is an indicator for neuronal network complexity; and the staining for activated caspases 3/7 serves as an apoptotic marker. These methodological differences between the assays could account for the differences in results. Nonetheless, our findings highlight that manipulation of SMAD2 expression can be a promising therapeutic approach against PD, since knockdown of *SMAD2* showed pronounced protective efficacy consistently across all three readout methods.

In this project, we identified an upregulation of *TGFB1*, *TGFB2*, *SMAD2*, and *SMAD3* as a consequence of aSyn-overexpression in LUHMES cells. In accordance with this, it has been previously shown that TGFb1 is upregulated in the striatum of post-mortem PD patients [46]. Furthermore, it has been reported previously that TGFb2 was elevated in the vCSF of PD patients in comparison to healthy controls [47]. The up-regulation of genes and proteins of the TGFb pathway in multiple regions of the brain could indicate a global up-regulation in the brains of PD patients. However, the expression patterns of the genes and proteins of the TGFb pathway in the brains of PD patients are not well understood. In particular, it is unknown whether such up-regulation follows the spread of aSyn pathology. Furthermore, the time point at which such an up-regulation could occur is unknown. Therefore, to understand if the up-regulation of genes and proteins of the TGFb pathway in the brains of PD patients is global and progressive, deeper analyses of various brain regions would be needed, which was beyond the scope of this study. From a broader perspective, and in comparison to previous works on the role of TGFb signaling on midbrain dopaminergic neurons, the present findings, which utilized LUHMES cells, emphasized the contrasting time-dependent impact of this pathway. While TGFb has an essential role in the development, survival, and maturation of dopaminergic neurons, at adult or aging stages, it does not seem to protect or maintain neurons even when these neurons are under stress or pathological conditions.

Our results offer valuable insights for the development of potential drugs against PD. As an example, we identified two inhibitors of GSK3B and one inhibitor of ALK5, which significantly protected dopaminergic neurons against aSyn-induced toxicity. Among them, tideglusib has already been used in clinical trials in patients with progressive supranuclear palsy [48] and Alzheimer’s disease [49], meaning existing data on its dosage and safety are already available. In the context of PD, tideglusib treatment protected dopaminergic neurons in mice against MPP^+^-induced toxicity [50]. Together with our findings that tideglusib protected dopaminergic neurons from aSyn-induced toxicity, it would be worthwhile to continue with clinical trials of tideglusib in PD. On the other hand, SM16 has been used in vivo in rodents to inhibit tumor growth, and therefore, its safety in vivo has also been proven [51]. However, SM16 has never been used in clinical trials or in experiments that involve non-human primates in vivo. As our results provided clear indications that the use of SM16 strongly protected dopaminergic neurons from aSyn-induced toxicity in vitro, it would be worthwhile to assess if SM16 also exerts its protectiveness against aSyn-induced toxicity in vivo [52].

In summary, our findings highlight the detrimental role of TGFb signaling in relation to aSyn-induced toxicity. Furthermore, we observed that the reduction of TGFb signaling protected against aSyn-induced toxicity. Our results also suggest that *SNCA* expression and TGFb pathway gene expression may influence each other. The alignment between our in vitro results and the published observations in human post-mortem brains as well as vCSF of PD patients [46, 47], supports our findings and thus could be the basis for the development of TGFb-inhibiting drugs as therapeutic approach for PD and related synucleinopathies.

## Materials and methods

### Cell culture

LUHMES cells (CRL-2927, American Type Culture Collection) were used in all in vitro experiments, as previously published by our group [9, 10]. Cells were kept at 37 °C, 5% CO_2_, and moisturized air. The cell culture flasks (Nunc, Thermo Fisher Scientific, Waltham, MA, USA) were pre-coated at 37 °C overnight with 0.1 mg/mL poly-L-ornithine (PLO; Sigma-Aldrich, St. Louis, MO, USA). For proliferation, LUHMES cells were maintained in proliferation medium (Dulbecco’s modified Eagle medium/nutrient mixture F-12, DMEM/F12 (Sigma-Aldrich) with 1% N2 supplement (Thermo Fisher Scientific), and 0.04 μg/mL fibroblast growth factor-basic, PeproTech, Rocky Hill, CT, USA). For the actual experiments, multi-well plates or flasks (Thermo Fisher Scientific) were pre-coated with 0.1 mg/mL PLO (37 °C, overnight) and subsequently with 5 µg/mL fibronectin (FN; R&D Systems, Minneapolis, MN, USA) at 37 °C overnight. To initiate the differentiation process, differentiation medium consisting of DMEM/F12 (Sigma-Aldrich) with 1% N2 supplement (Thermo Fisher Scientific), 1 μg/mL tetracycline (Sigma-Aldrich), 0.49 mg/mL dibutyryl-cyclic adenosine monophosphate (Sigma-Aldrich), and 2 ng/μL glial cell line-derived neurotrophic factor (R&D Systems) was used [53]. Prior to the coating with FN, multi-well plates or flasks were washed three times with sterile water to remove excess PLO. Before seeding, the multi-well plates or flasks were washed three times with sterile phosphate-buffered saline (PBS; Sigma-Aldrich) to remove excess FN. Cell cultures are routinely tested for mycoplasma to ensure they are not contaminated.

### Adenoviral transduction

On days in vitro (DIV) 2, cells were transduced with adenoviral vectors (Charles Rivers Laboratories, Leiden, the Netherlands) to overexpress human wild-type aSyn or GFP under a cytomegalovirus promoter. 24 h post-transduction, cells were washed three times with Hank’s balanced salt solution (HBSS; Sigma-Aldrich) to remove residual virus and were then replenished with fresh DM, as previously described [53, 54].

### siRNA transfection/ligand and inhibitor treatment

Approximately 4–6 h after the removal of viral vectors, cells were transfected with siRNAs and/or were treated with ligands or inhibitors. Two different types of siRNAs were used, namely MISSION^®^ endoribonuclease-prepared siRNA (esiRNA, Sigma-Aldrich) and siPOOLs siRNAs (siTOOLs, Martinsried, Germany). Before transfection, the siRNAs were mixed with Opti-MEM™ medium (Thermo Fisher Scientific) and the transfection reagent Lipofectamine™ RNAiMax (Thermo Fisher Scientific). The mixture was then vortexed and incubated for 20 minutes (min) prior to treating the cells. The final concentrations of the esiRNA were 200 ng/µL and those of siPOOL were 5 nM. Ligands or inhibitors were diluted in DM into their respective final concentrations immediately before treating the cells.

The following ligands and inhibitors were used: Recombinant human TGFb1 protein (R&D Systems), recombinant human TGFb2 protein (R&D Systems), recombinant human TGFb3 protein (R&D Systems), tideglusib (Sigma-Aldrich), GSK3B-inhibitor VIII (Sigma-Aldrich), or SM16 (Tocris, Minneapolis, MN, USA)

### Lactate dehydrogenase cytotoxicity assay

Lactate dehydrogenase (LDH), as a measure for cytotoxicity, was quantified by spectrophotometric measurement of the absorbance at 340 nm, which is an indirect measurement of the consumption of nicotinamide adenine dinucleotide (NADH) by LDH. Briefly, culture medium was harvested from cells on DIV8, and was mixed with a reaction buffer consisting of 74.24 mM Tris/HCl (Sigma-Aldrich), 185.6 mM NaCl (Sigma-Aldrich), 3.2 mM pyruvate (Sigma-Aldrich), and 4 mM NADH (Sigma-Aldrich) in water. We then measured the absorbance of the mixture at 340 and 420 nm with the use of a plate reader (FLUOstar Omega, BMG Labtech, Ortenberg, Germany). Medium from cells lysed with 10% Triton X-100 (Sigma-Aldrich) 1 h before the assay was used as a positive control. Unless otherwise specified, all LDH data were normalized to the LDH measured in aSyn-overexpressing cells.

### Real-time quantitative polymerase chain reaction

We used RNeasy^®^ Mini kit (Qiagen, Hilden, Germany) for the extraction of RNA. Briefly, we harvested cells on DIV6 using RLT buffer (activated with 1% beta-mercaptoethanol; Sigma-Aldrich) and cell scrapers. Then, the samples were run through genomic DNA eliminator columns. The resultant flow-through was mixed thoroughly with 70% ethanol, and the mixture was eluted through RNA spin-columns. RNase-free water was used to dissolve the RNA extracted from the RNA spin-columns. RNA concentration was determined using a NanoDrop™ 2000 Microvolume Spectrophotometer (Thermo Fisher Scientific). Reverse transcription was performed using the iScript™ cDNA Synthesis kit (Bio-Rad Laboratories, Hercules, CA, USA). A CFX96 Touch™ Real-Time PCR Detection System (Bio-Rad Laboratories) was used for the real-time quantitative polymerase chain reaction (qPCR), and SYBR™ Select Master Mix for CFX (Thermo Fisher Scientific) was used as a dye to indicate double-stranded DNA formed in the qPCR process.

### Western blotting

Cells were harvested on DIV6 using M-PER™ lysis buffer (Thermo Fisher Scientific) supplemented with protease and phosphatase inhibitors (Roche, Basel, Switzerland). The lysates were then centrifuged at 4 °C, 10 min, 13,000×*g* to remove cell debris. The concentration of each lysate was then determined by performing the bicinchoninic acid (BCA) assay using the Pierce™ BCA protein assay kit (Thermo Fisher Scientific). Then, protein samples were loaded on 4–12% Criterion™ XT bis-tris precast protein gels (Bio-Rad Laboratories) with 2-(N-morpholino)ethanesulfonic acid (MES) running buffer. After the sodium dodecyl sulfate–polyacrylamide gel electrophoresis (SDS–PAGE), proteins in the gels were transferred onto pre-assembled 0.2 µm polyvinylidene fluoride (PVDF) membranes (Bio-Rad Laboratories) in a Trans-Blot^®^ SD semi-dry blotter (Bio-Rad Laboratories). After the transfer, membranes were incubated with 0.4% paraformaldehyde (PFA; Sigma-Aldrich) for 30 min. The membranes were then rinsed with PBS three times (5 min each), followed by an incubation in blocking solution (30% Roti^®^Block (Carl Roth, Karlsruhe, Germany)) in Tris-Buffered Saline with 0.1% Tween 20 (TBST; Sigma-Aldrich) for 1 h at room temperature (RT). Then, the membranes were incubated with primary antibodies diluted with 10% Roti^®^Block (Carl Roth)/TBST solution at 4 °C overnight. The next day, membranes were rinsed three times with TBST (5 min each) to remove the primary antibodies and then were incubated with the secondary antibodies (diluted in TBST) for 2 h at RT. After the incubation with the secondary antibodies, the membranes were rinsed three times with TBST (5 min each) to remove the secondary antibodies. Then, the membranes were incubated with Clarity™ Western ECL Substrate (Bio-Rad Laboratories). An Odyssey^®^ Fc Imager (LI-COR Biotechnology, Lincoln, NE, USA) was used to obtain images of the membranes.

The following primary antibodies were used: rabbit anti-aSyn (1:500; 701085, Thermo Fisher Scientific); rabbit anti-GAPDH (1:1000; 2118, Cell Signaling Technology, Danvers, MA, USA); rabbit anti-GSK3B (1:5000; ab32391, Abcam, Cambridge, UK). The following secondary antibodies were used: HRP-coupled goat anti-mouse IgG (1:5000; PI-2000, Vector Laboratories, Burlingame, CA, USA); HRP-coupled goat anti-rabbit IgG (1:5000; PI-1000, Vector Laboratories).

### Immunocytochemistry

Prior to the start of experiments, glass cover slips (Bellco Glass Inc., Vineland, NJ, USA) were coated in 24-well plates (Thermo Fisher Scientific) with PLO and FN before seeding the cells. The coating procedures and the cell culture were performed as described above. On DIV8, cells were washed two times with HBSS before fixation with 4% PFA in PBS. The fixation was performed at RT for 45 min. Then, the cells were incubated with 0.1% Triton X-100 (Sigma-Aldrich) solution for 15 min at RT. Afterwards, cells were washed three times with PBS, followed by an incubation in blocking solution (5% normal horse serum in PBS) for 1 h at RT. Then, primary antibodies, diluted in blocking solution, were added to the cells, followed by an incubation overnight at 4 °C. The primary antibodies were removed on the next day, and the cells were washed three times with PBS. Then, the cells were incubated with the secondary antibodies (diluted in blocking solution) for 2 h at RT in the dark to prevent photobleaching. The cells were then washed once with PBS, and were subsequently incubated with 4’,6-diamidino-2-phenylindole (DAPI; Sigma-Aldrich, diluted with PBS at a concentration of 1:10,000) in the dark for 20 min at RT. Before mounting, cells were washed three times with PBS to remove all the antibodies and DAPI. Mowiol^®^ (Carl Roth) was used as a mounting medium. Images were acquired with the use of a Leica DMi8 fluorescence microscope (Leica, Wetzlar, Germany) with ×40 magnification. Images were processed using the Leica Application Suite X software. To quantify the density of the neuronal network, we counted the number of quadruple points per cell by using a modified Neurite Analyzer plugin on the Fiji software [55]. Each quadruple point is defined as a neuronal junction that contains, or was connected to, four different neurites.

The following primary antibodies were used: rabbit anti-aSyn (1:1000; 701085, Thermo Fisher Scientific); mouse anti-beta (III)-tubulin (1:1000; 801202, BioLegend, San Diego, CA, USA). The following secondary antibodies were used: Alexa Fluor 555 Donkey anti-rabbit IgG (1:500; A31572, Thermo Fisher Scientific); Alexa Fluor 647 Donkey anti-mouse IgG (1:500; A32794, Thermo Fisher Scientific).

### Detection of activated caspase-3/7 in apoptotic cells

CellEvent™ Caspase-3/7 Detection Reagent (Thermo Fisher) was used to detect the activation of caspase 3/7 in apoptotic cells. Briefly, the cell culture was performed as mentioned previously. On DIV8, prior to the assay, the cell culture medium was removed from the cells. CellEvent™ reagent was diluted with pre-warmed HBSS to a final working concentration of 2 µM. The diluted CellEvent™ reagent was added to the cells in a dropwise manner to prevent the cell layer from detaching. The cells were incubated with the reagent for 30 min in the cell culture incubator (37 °C, 5% CO_2_). After 30 min, the diluted CellEvent™ solution was replaced by diluted Hoechst/HBSS solution (final concentration: 1:10,000) and was incubated in the cell culture incubator for 5 min. Imaging was performed with a DMi8 live cell imaging fluorescence microscope. The images were processed using the Leica Application Suite X software. The activated caspases signal in the area of the nuclei was quantified by using the Fiji software.

### Statistical analysis

Data were tested for normality and were analyzed by GraphPad Prism (version 10, GraphPad Software, La Jolla, CA, USA). In all experiments, a minimum of three individual biological replicates were performed. Data were analyzed by multiple *t*-test if only two experimental groups were present, or by analysis of variance (ANOVA) followed by a post-hoc Bonferroni’s test, if at least three experimental groups were present. Statistical significance was assumed if *p*-values were lower than 0.05. All graphs are presented with error bars indicating the standard error of the mean (SEM). Investigators were not blinded to group allocation during the experiments or outcome assessments. No statistical method was employed to predetermine sample size; instead, sample sizes were determined empirically based on prior experience and observed experimental variability.

## Supplementary information

Supplementary Figures and Legends

Full Western blot images, uncropped

## Data Availability

All datasets used and analyzed in this study are available from the corresponding authors on reasonable request.
