# Supplementary material for: Inhibition of TGF-beta signaling protects from alpha-synuclein induced toxicity
Source: Cell Death Discov. 2025 Dec 12;12:44. doi: 10.1038/s41420-025-02901-2 (PMC12830698; doi:10.1038/s41420-025-02901-2)
Supplement: Supplementary file 1 — Supplementary Figures and Legends [file 41420_2025_2901_MOESM1_ESM.docx]

**
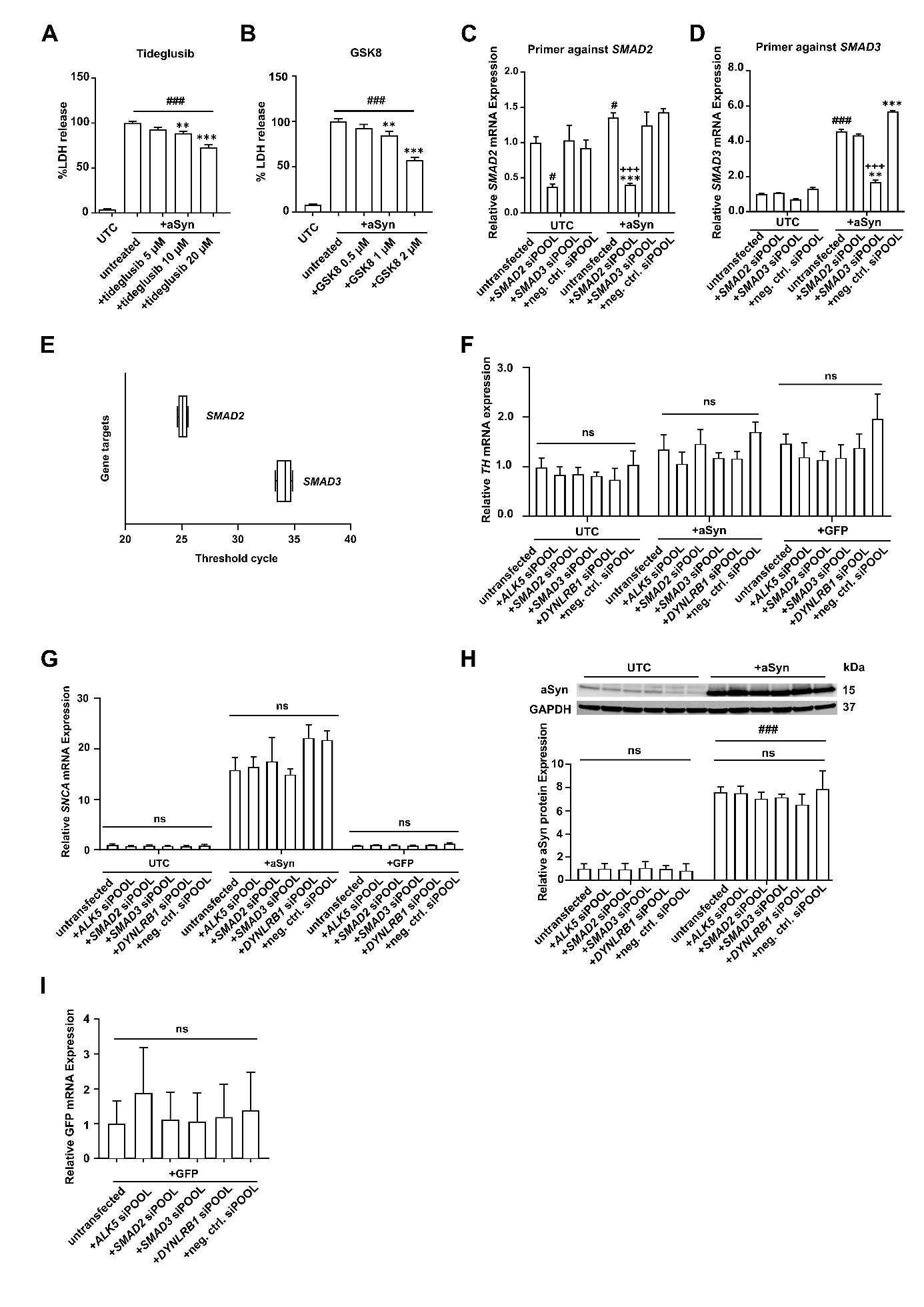
**

**Supplementary Fig. S1.**

**A**: Quantification of LDH release into the cell culture medium after treatment of tideglusib in aSyn-overexpressing cells. Tideglusib, a GSK3B inhibitor, significantly protected aSyn-overexpressing cells at concentrations of 10 µM and 20 µM.

**B**: Quantification of LDH release into the cell culture medium showed that the treatment of GSK3B inhibitor-VIII (GSK8) significantly protected aSyn-overexpressing cells at concentrations of 1 µM and 2 µM.

**C:** Quantification of the relative mRNA expression of *SMAD2* after transfection with the siPOOL siRNA against *SMAD2*. The siPOOL siRNA against *SMAD2* led to a knockdown of *SMAD2* in both untransduced cells (UTCs) and aSyn-overexpressing cells. The expression of *SMAD2* was significantly up-regulated upon aSyn-overexpression.

**D**: Quantification of the relative mRNA expression of *SMAD3* after transfection with the siPOOL siRNA against *SMAD3*. The siPOOL siRNA against *SMAD3* led to a knockdown of *SMAD3* in both UTCs and aSyn-overexpressing cells. The expression of *SMAD3* was significantly up-regulated upon aSyn-overexpression.

**E:** The plot shows number of threshold cycles in the qPCR until which expression of the respective genes was detected in untransduced LUHMES cells. The expression of *SMAD2* was between 23^rd^ and 26^th^ threshold cycle. The expression of *SMAD3* was much lower in comparison to *SMAD2* in the chart, reaching the threshold value ~10 cycles later.

**F**: Quantification of the relative mRNA expression of *TH* (tyrosine hydroxylase) after transfection with the siPOOL siRNA against *ALK5*, *SMAD2*, *SMAD3*, or *DYNLRB1* did not significant alter the expression of *TH* in UTCs, aSyn-overexpressing cells or GFP-expressing cells.

**G**: Quantification of the relative mRNA expression of *SNCA* (gene that encodes aSyn) after transfection with the siPOOL siRNA against *ALK5*, *SMAD2*, *SMAD3*, or *DYNLRB1* did not significant alter the expression of *SNCA* in UTCs, aSyn-overexpressing cells or GFP-expressing cells.

**H**: Representative Western blots with an antibody against aSyn confirmed that the knockdown of *ALK5*, *SMAD2*, *SMAD3*, and *DYNLRB1* by siPOOL siRNA did not lead to a significant change in the expression of aSyn protein in both UTCs and aSyn-overexpressing cells. Full size blot images can be found in Supplementary Fig. S1C.

**I**: Quantification of the relative mRNA expression of GFP in GFP-expressing cells after the transfection with the siPOOL siRNA against *ALK5*, *SMAD2*, *SMAD3*, or *DYNLRB1*. The expression of GFP was not altered by the knockdown of *ALK5*, *SMAD2*, *SMAD3*, or *DYNLRB1*.

UTCs: untransduced cells; aSyn/+aSyn: cells overexpressing alpha-Synuclein; +GFP: cells expressing GFP; GSK8: GSK3B inhibitor-VIII*; ALK5* siPOOL: siPOOL siRNA against *ALK5*; neg. ctrl. siPOOL: negative control siPOOL siRNA; *SMAD2* siPOOL: siPOOL siRNA against *SMAD2*; *SMAD3* siPOOL: siPOOL siRNA against *SMAD3*; *DYNLRB1* siPOOL: siPOOL siRNA against *DYNLRB1*; untreated: cells that were not treated with SM16; untransfected: cells that were not transfected with siPOOL siRNAs.

#: *p*<0.05, ###: *p*<0.001 against untransfected UTCs; **: *p*<0.01, ***: *p*<0.001 against untransfected aSyn-overexpressing cells; +++: *p*<0.001 against aSyn + neg. ctrl. siPOOL; ns: not significant.


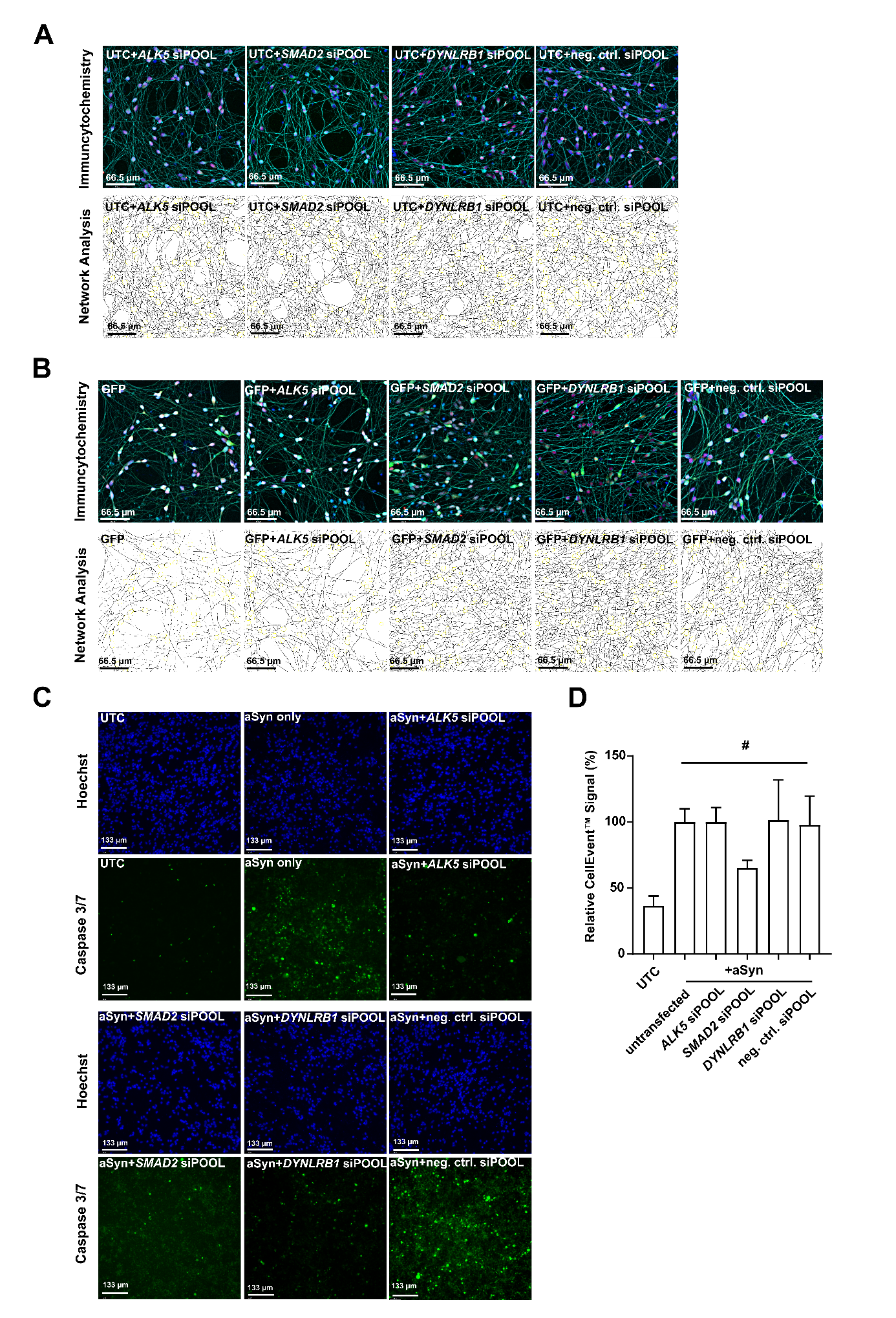


**Supplementary Fig. S2**

**A, B:** Representative images of the ICC staining against DAPI (blue), GFP (green, if any), aSyn (red), and beta-III tubulin (cyan) in untransduced cells (UTCs; **A)** and GFP-expressing cells **(B)**. Corresponding network analysis for each representative figure is shown below the staining, in which black lines outlined the neurites and the yellow lines outlined the nuclei.

**C:** Representative images of the staining of activated caspases 3/7 (CellEvent™ Caspase-3/7 Detection Reagent; green) and Hoechst (blue).

**D:** Quantification plot of (C). The knockdown of *SMAD2* in aSyn-overexpressing cells led to an obvious reduction in the activated caspases-3/7-positive signal.

UTCs: untransduced cells; +aSyn: cells overexpressing aSyn; +GFP: cells expressing GFP; *ALK5* siPOOL: siPOOL siRNA against *ALK5*; neg. ctrl. siPOOL: negative control siPOOL siRNA; *SMAD2* siPOOL: siPOOL siRNA against *SMAD2*; *DYNLRB1* siPOOL: siPOOL siRNA against *DYNLRB1*.


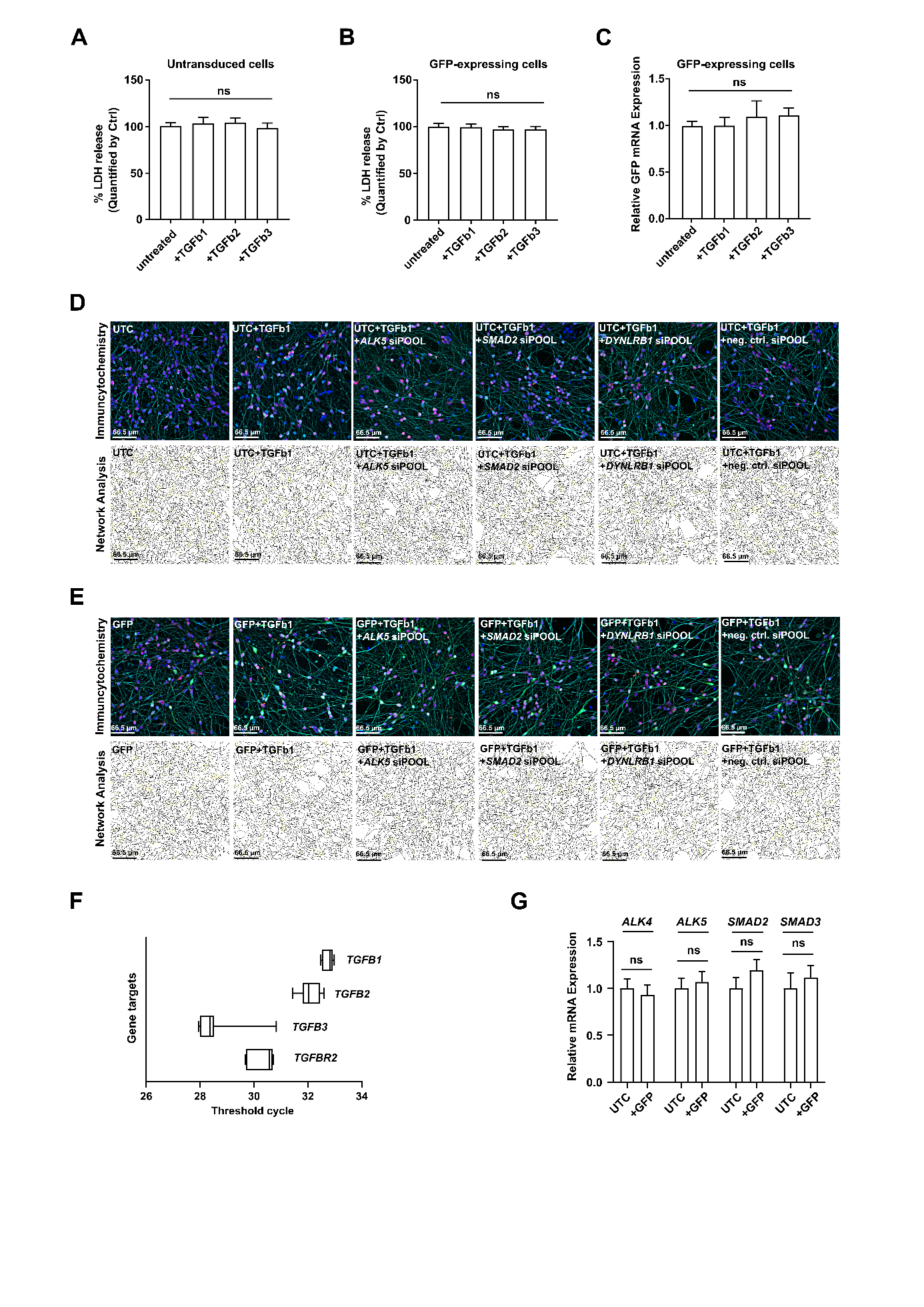


**Supplementary Fig. S3**

**A, B**: Quantification of LDH release into the cell culture medium after treatment with TGFb1, TGFb2, or TGFb3 in untransduced cells (UTCs; **A**) and GFP-expressing cells **(B)**. Treatment of TGFb ligands did not lead to changes in cell viability in both UTCs and GFP-expressing cells.

**C:** Quantification of the relative mRNA expression of GFP in GFP-expressing cells after the treatment with TGFb1, TGFb2, or TGFb3. Treatment of TGFb ligands did not lead to changes in the expression of GFP.

**D, E:** Representative images of the ICC staining against DAPI (blue), GFP (green, if any), aSyn (red), and beta-III tubulin (cyan) in UTCs **(D)** and GFP-expressing cells **(E)**. Corresponding network analysis for each representative figure is shown below the staining, in which black lines outlined the neurites and yellow lines outlined the nuclei.

**F**: The plot shows the number of threshold cycles in the qPCR until which expression of the respective genes was detected in untransduced LUHMES cells. The expression of *TGFB1*, *TGFB2*, *TGFB3,* and *TGFBR2* were comparable (between 28^th^ and 32^nd^ threshold cycle).

**G**: Quantification of the relative mRNA expression of *ALK4*, *ALK5*, *SMAD2*, and *SMAD3* in UTCs and GFP-expressing cells. The expression of *ALK4*, *ALK5*, *SMAD2*, and *SMAD3* was not affected by the expression of GFP, as compared to UTCs.

UTCs: untransduced cells; +GFP: cells expressing GFP; *ALK5* siPOOL: siPOOL siRNA against *ALK5*; neg. ctrl. siPOOL: negative control siPOOL siRNA; *SMAD2* siPOOL: siPOOL siRNA against *SMAD2*; *DYNLRB1* siPOOL: siPOOL siRNA against *DYNLRB1*; untreated: cells that were not treated with TGFb ligands.

ns: no statistical significance

**
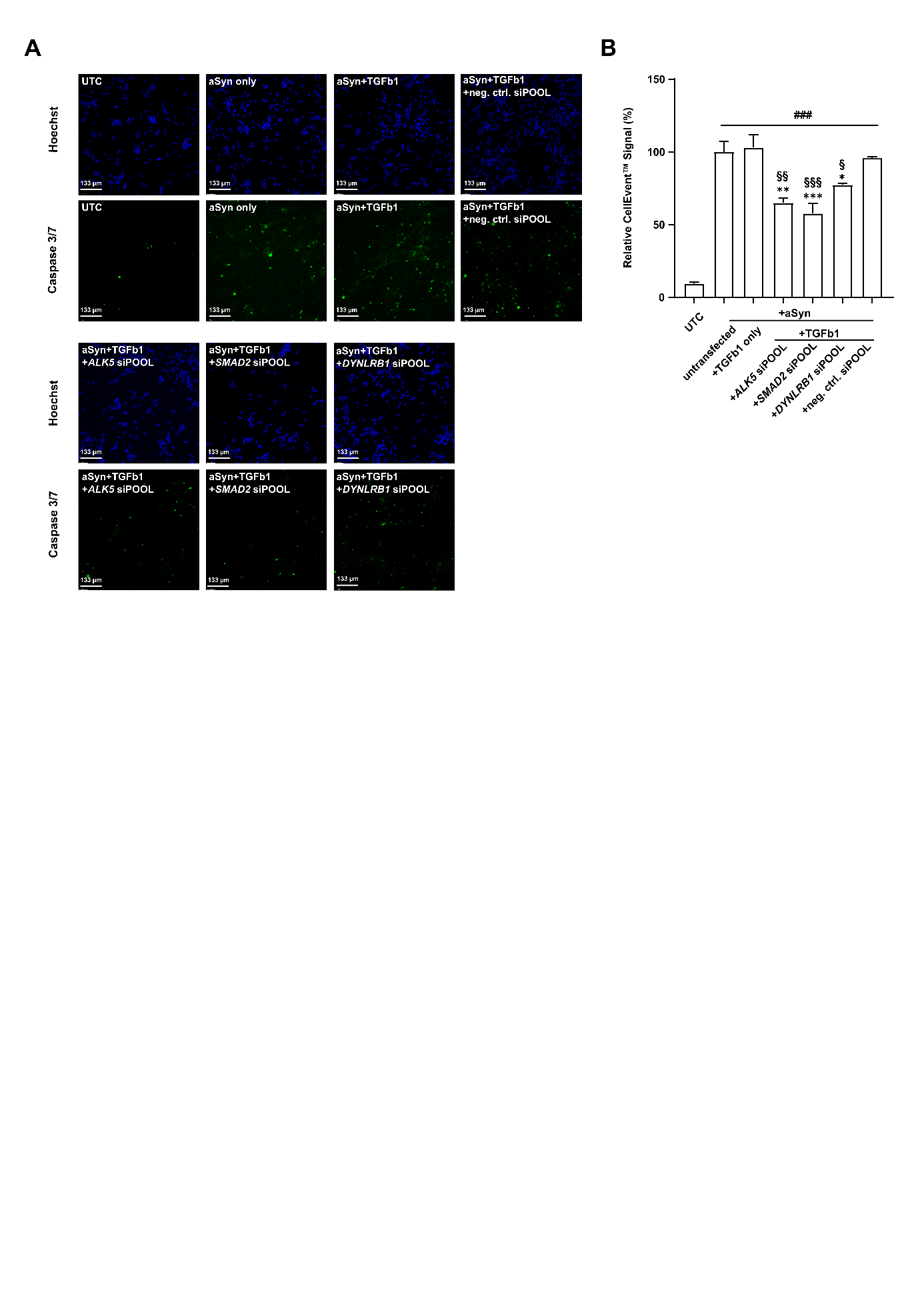
Supplementary Fig. S4**

**A**: Representative images of the staining of activated caspases 3/7 (CellEvent™ Caspase-3/7 Detection Reagent;green) and Hoechst (blue).

**B**: Quantification plot of (A). The knockdown of ALK5, *SMAD2*, and DYNLRB1 in TGFb1-treated aSyn-overexpressing cells led to a significant reduction in the activated caspases-3/7-positive signal.

**C**: Full-size uncropped Western blot images showing the protein expression of aSyn (above) and GAPDH (below) after the treatment of TGFb1, TGFb2, or TGFb3 in UTCs, aSyn-overexpressing cells, and GFP-expressing cells. In contrast to our qPCR data (Fig. 5F), the treatment of TGFb ligands did not lead to a statistically significant increase in aSyn expression in aSyn-overexpressing cells.

UTCs: untransduced cells; +aSyn: cells overexpressing aSyn; *ALK5* siPOOL: siPOOL siRNA against *ALK5*; neg. ctrl. siPOOL: negative control siPOOL siRNA; *SMAD2* siPOOL: siPOOL siRNA against *SMAD2*; *DYNLRB1* siPOOL: siPOOL siRNA against *DYNLRB1*.

*: *p* <0.05, **: *p* <0.01, ***: *p* <0.001 against untreated aSyn; §: *p* <0.05, §§§: *p* <0.001 against aSyn+TGFb ligand.
