# Supplementary material for: Inhibition of TGF-beta signaling protects from alpha-synuclein induced toxicity
Source: Cell Death Discov. 2025 Dec 12;12:44. doi: 10.1038/s41420-025-02901-2 (PMC12830698; doi:10.1038/s41420-025-02901-2)
Supplement: Supplementary file 2 — Full Western blot images, uncropped [file 41420_2025_2901_MOESM2_ESM.docx]

**A:** Full-size Western blot images of Fig. 2C.

**B:** Full-size Western blot images of Fig. 2D.

UTCs: untransduced cells; aSyn/+aSyn: cells overexpressing alpha-Synuclein; *GSK3B* esiRNA: esiRNA against *GSK3B*; *GSK3B* siPOOL: siPOOL siRNA against *GSK3B*; neg. ctrl. esiRNA: negative control esiRNA; neg. ctrl. siPOOL: negative control siPOOL siRNA; untransfected: cells that were not transfected with esiRNAs or siPOOL siRNAs.





**C:** Full-size Western blot images of Supplementary Fig. S1H.

UTCs: untransduced cells; aSyn/+aSyn: cells overexpressing alpha-Synuclein; *ALK5* siPOOL: siPOOL siRNA against *ALK5*; neg. ctrl. siPOOL: negative control siPOOL siRNA; *SMAD2* siPOOL: siPOOL siRNA against *SMAD2*; *SMAD3* siPOOL: siPOOL siRNA against *SMAD3*; *DYNLRB1* siPOOL: siPOOL siRNA against *DYNLRB1*; untransfected: cells that were not transfected with siPOOL siRNAs.
